# Sexual Trauma, Polygenic Scores, and Mental Health Diagnoses and Outcomes

**DOI:** 10.1001/jamapsychiatry.2024.3426

**Published:** 2024-10-30

**Authors:** Allison M. Lake, Yu Zhou, Bo Wang, Ky’Era V. Actkins, Yingzhe Zhang, John P. Shelley, Anindita Rajamani, Michael Steigman, Chris J. Kennedy, Jordan W. Smoller, Karmel W. Choi, Nikhil K. Khankari, Lea K. Davis

**Affiliations:** 1 Division of Genetic Medicine, Department of Medicine, Vanderbilt University Medical Center, Nashville, Tennessee; 2 Vanderbilt Genetics Institute, Vanderbilt University Medical Center, Nashville, Tennessee; 3 Center for Precision Psychiatry, Department of Psychiatry, Massachusetts General Hospital, Boston; 4 Psychiatric and Neurodevelopmental Genetics Unit, Center for Genomic Medicine, Massachusetts General Hospital, Boston; 5 Department of Epidemiology, Harvard T.H. Chan School of Public Health, Boston, Massachusetts; 6 Department of Biomedical Informatics, Vanderbilt University Medical Center, Nashville, Tennessee; 7 Department of Computer Science and Engineering, University of Minnesota, Minneapolis; 8 Division of Data-Driven and Digital Medicine, Department of Medicine, Icahn School of Medicine at Mount Sinai, New York, New York

## Abstract

**Importance:**

Leveraging real-world clinical biobanks to investigate the associations between genetic and environmental risk factors for mental illness may help direct clinical screening efforts and evaluate the portability of polygenic scores across environmental contexts.

**Objective:**

To examine the associations between sexual trauma, polygenic liability to mental health outcomes, and clinical diagnoses of schizophrenia, bipolar disorder, and major depressive disorder in a clinical biobank setting.

**Design, Setting, and Participants:**

This genetic association study was conducted using clinical and genotyping data from 96 002 participants across hospital-linked biobanks located at Vanderbilt University Medical Center (VUMC), Nashville, Tennessee (including 58 262 individuals with high genetic similarity to the 1000 Genomes Project [1KG] Northern European from Utah reference population [1KG-EU-clustered] and 11 047 with high genetic similarity to the 1KG African-ancestry reference population of Yoruba in Ibadan, Nigeria [1KG-YRI-clustered]), and Mass General Brigham (MGB), Boston, Massachusetts (26 693 individuals with high genetic similarity to the combined European-ancestry superpopulation [1KG-EU-clustered]). Clinical data analyzed included diagnostic billing codes and clinical notes spanning from 1976 to 2023. Data analysis was performed from 2022 to 2024.

**Exposures:**

Clinically documented sexual trauma disclosures and polygenic scores for schizophrenia, bipolar disorder, and major depressive disorder.

**Main Outcomes and Measures:**

Diagnoses of schizophrenia, bipolar disorder, and major depressive disorder, determined by aggregating related diagnostic billing codes, were the dependent variables in logistic regression models including sexual trauma disclosure status, polygenic scores, and their interactions as the independent variables.

**Results:**

Across the VUMC and MGB biobanks, 96 002 individuals were included in analyses (VUMC 1KG-EU-clustered: 33 011 [56.7%] female; median [range] age, 56.8 [10.0 to >89] years; MGB 1KG-EU-clustered: 14 647 [54.9%] female; median [range] age, 58.0 [10.0 to >89] years; VUMC 1KG-YRI-clustered: 6961 [63.0%] female; median [range] age, 44.6 [10.1 to >89] years). Sexual trauma history was associated with all mental health conditions across institutions (ORs ranged from 8.83 [95% CI, 5.50-14.18] for schizophrenia in the VUMC 1KG-YRI-clustered cohort to 17.65 [95% CI, 12.77-24.40] for schizophrenia in the VUMC 1KG-EU-clustered cohort). Sexual trauma history and polygenic scores jointly explained 3.8% to 8.8% of mental health phenotypic variance. Schizophrenia and bipolar disorder polygenic scores had greater associations with mental health outcomes in individuals with no documented disclosures of sexual trauma (schizophrenia interaction: OR, 0.70 [95% CI, 0.56-0.88]; bipolar disorder interaction: OR, 0.83 [95% CI, 0.74-0.94]).

**Conclusions and Relevance:**

Sexual trauma and mental health polygenic scores, while correlated with one another, were independent and joint risk factors for severe mental illness in a large, diverse hospital biobank population. Furthermore, associations of schizophrenia and bipolar disorder polygenic scores with respective diagnoses were greater in those without disclosures, suggesting that genetic predisposition to mental illness as measured by polygenic scores may be less impactful in the presence of this severe environmental risk factor.

## Key Points

**Question:**

What are the independent, joint, and interacting contributions of sexual trauma history and polygenic liability to the risk of schizophrenia, bipolar disorder, and major depressive disorder diagnoses?

**Findings:**

This genetic association study identified associations between sexual trauma and mental health diagnoses in 96 002 patients from 2 geographically distinct academic medical center settings, with both sexual trauma history and polygenic scores showing significant independent and joint associations with schizophrenia and major depressive disorder across all analyses. In a cross-site meta-analysis, polygenic scores for schizophrenia and bipolar disorder demonstrated an interaction with sexual trauma disclosures in which the association between each polygenic score and its respective diagnosis was weaker among individuals reporting sexual trauma.

**Meaning:**

Sexual trauma and polygenic scores were independently and jointly important risk factors for mental health conditions, yet the associations of polygenic scores generated using existing methods may be less impactful than the mental health risk conferred by sexual trauma among patients with this history.

## Teaser

This genetic association study examined the associations between sexual trauma, polygenic liability to mental health outcomes, and clinical diagnoses of schizophrenia, bipolar disorder, and major depressive disorder in a clinical biobank setting.

## Introduction

Sexual trauma, encompassing sexual abuse, assault, and rape, is an important public health concern with wide-ranging physical and mental health consequences, affecting 25% to 58% of people of varying gender identities in the United States.^1,2^ Epidemiological evidence points to childhood trauma broadly and sexual trauma specifically as risk factors for psychiatric outcomes, including major depressive disorder (MDD), bipolar disorder (BD), and schizophrenia.^3,4,5,6,7,8,9,10^ Additionally, each of these conditions has a modest (MDD) to strong (BD, schizophrenia) genetic component.^11,12,13^ Thus, a growing awareness of the importance of social and environmental risk factors in the field of psychiatric genetics has led to an increasing number of investigations into the joint influences of genetics and adverse experiences on psychiatric illness liability. Molecular genetic studies of gene-by-environment interactions have historically focused on candidate genes and variants,^14,15,16^ while recent studies have examined gene-by-environment interactions in mental health using whole-genome variance components approaches^17,18^ or polygenic scores (PGSs).^19,20,21,22,23,24,25,26,27,28,29,30,31,32,33,34^ Gene-by-environment interaction analyses using PGS in BD have been limited to case-only studies,^35,36^ highlighting a need for further investigation of this phenotype. Several studies have examined the interactions between childhood trauma and PGS in schizophrenia, with conflicting results,^27,37,38,39^ sometimes owing to small sample size.^29^ Studies of gene-by-environment interactions in MDD, while better powered, have also shown conflicting results, with observations of positive associations,^19,21,23,28,30^ negative associations,^20,30^ and nonsignificant findings^22,25,26^ between gene-by-environment interactions and depression diagnosis. Notably, Peyrot and colleagues^22^ were unable to replicate previous gene-by-environment results using newer genome-wide association study (GWAS) summary statistics, illustrating the need for replication and meta-analysis of gene-by-environment interaction findings across diverse cohorts.

Studying gene-by-environment interactions in psychiatry is important for both improving etiological knowledge and identifying high-risk groups for early intervention. Furthermore, as PGSs are being considered for future use in clinical settings,^40,41^ it is important to evaluate the associations between PGSs and psychiatric outcomes across different environmental contexts. However, to date, no study to our knowledge has systematically evaluated the joint influences of genetics and trauma across multiple mental health conditions in a real-world clinical setting. Furthermore, prior studies have been primarily limited to European-ancestry populations. Here, we leverage electronic health records (EHRs) linked to genomic data from 2 large hospital systems that are part of PsycheMERGE, a cross-institutional collaborative network of EHR-linked biobanks with a focus on advancing precision psychiatry in diverse populations. Leveraging PsycheMERGE and an existing phenotyping approach to detecting sexual trauma disclosures from clinical notes,^8^ we conducted multiancestry genetic analysis examining the influences of sexual trauma, PGS, and their interactions in schizophrenia, BD, and MDD.

## Methods

### Participants

This study was conducted in 2 EHR-linked biobanks in the PsycheMERGE Network, located at Vanderbilt University Medical Center (VUMC), Nashville, Tennessee, and Mass General Brigham (MGB), Boston, Massachusetts, academic medical centers in the southeastern and northeastern United States, respectively. The study included 96 002 individuals receiving care from 1976 to 2023 at VUMC or MGB with available genotyping and clinical data. Each study received ethical approval by the VUMC or MGB institutional review board, including an informed consent waiver for the use of retrospective medical record data with no patient interaction. All participants provided written informed consent for inclusion in the respective biobanks for broad-based research. Participants were at least 18 years old at the time of analysis and were required to have a median age at visit of at least 10 years due to the typical emergence of schizophrenia, BD, and MDD during adolescence or adulthood. Participants were required to meet a data floor of at least 3 unique visit dates across their EHR to mitigate nonrandom missing data between cases and controls (additional MGB criteria are described in the eMethods in Supplement 1). Additional sample details can be found in Table 1 and in the eMethods and eTable 1 in Supplement 1. This study followed the Strengthening the Reporting of Genetic Association Studies (STREGA) reporting guideline. Data analysis was performed from 2022 to 2024.

**Table 1.** Sample Characteristics

Characteristic	1KG-EU-clustered		VUMC 1KG-YRI-clustered (n = 11 047)
	VUMC (n = 58 262)	MGB (n = 26 693)	
EHR-recorded sex, No. (%)			
Female	33 011 (56.7)	14 647 (54.9)	6961 (63.0)
Male	25 251 (43.3)	12 046 (45.1)	4086 (37.0)
ST disclosure, No. (%)			
ST	844 (1.4)	752 (2.8)	238 (2.2)
No ST	57 418 (98.6)	25 941 (97.2)	10 809 (97.8)
EHR-median age, y^a^			
Mean (SD)	54.3 (18.3)	55.5 (16.3)	44.5 (18.4)
Median (range)	56.8 (10.0 to >89)	58.0 (10.0 to >89)	44.6 (10.1 to >89)
Unique days, No.^b^			
Mean (SD)	85.8 (101.9)	174.7 (179.5)	79.0 (107.8)
Median (range)	51.0 (3.0-2052.0)	120.0 (3.0-2503.0)	42.0 (3.0-1228.0)
Record length, y			
Mean (SD)	11.4 (7.5)	14.9 (7.4)	11.2 (7.7)
Median (range)	11.3 (0.0-32.8)	15.7 (0.1-24.4)	10.5 (0.0-32.7)
Schizophrenia, No. (%)			
Yes	274 (0.5)	255 (1.0)	182 (1.6)
No	57 845 (99.3)	26 321 (98.6)	10 820 (97.9)
Excluded^c^	143 (0.2)	117 (0.4)	45 (0.4)
Bipolar disorder, No. (%)			
Yes	1966 (3.4)	1449 (5.4)	405 (3.7)
No	55 242 (94.8)	24 770 (92.8)	10 445 (94.6)
Excluded^c^	1054 (1.8)	474 (1.8)	197 (1.8)
Major depressive disorder, No. (%)			
Yes	5965 (10.2)	5849 (21.9)	1026 (9.3)
No	49 505 (85.0)	19 401 (72.7)	9501 (86.0)
Excluded^c^	2792 (4.8)	1443 (5.4)	520 (4.7)
Substance use disorder, No. (%)			
Yes	11 035 (18.9)	7556 (28.3)	1986 (18.0)
No	47 227 (81.1)	19 137 (71.7)	9061 (82.0)
Inadequate housing, No. (%)			
Yes	1276 (2.2)	519 (1.9)	221 (2.0)
No	56 986 (97.8)	26 174 (98.1)	10 826 (98.0)

Abbreviations: EHR, electronic health record; MGB, Mass General Brigham; ST, sexual trauma; VUMC, Vanderbilt University Medical Center; 1KG-EU-clustered, 1000 Genomes Project European–clustered; 1KG-YRI-clustered, 1000 Genomes Project Yoruba in Ibadan, Nigeria–clustered.

^a^ EHR-median age refers to the median age at visit across all diagnostic codes in an individual’s record.

^b^ Unique days refers to the total number of distinct visit dates associated with diagnostic codes.

^c^ For a given diagnosis, individuals with a single component code but not meeting the full case definition (≥2 distinct code dates) were excluded from the statistical analyses of that diagnosis.

### Sexual Trauma Disclosures

We previously developed a phenotyping algorithm to detect disclosures of sexual trauma in clinical notes, using matches to specific key phrases^8^ (see the eMethods, eTable 2, and eTable 3 in Supplement 1). A total of 1842 individuals with sexual trauma disclosures were identified using this algorithm (2% of the full study sample; Table 1).

Performance metrics were calculated based on manual review of 100 randomly selected records with detected disclosures (50 from each site; eTable 4 in Supplement 1). Additional record review was conducted at VUMC to determine whether the trauma occurred during childhood or adulthood (eTable 5 in Supplement 1). Finally, at VUMC, an additional 73 records (25 individuals with both a detected disclosure and a diagnosis of schizophrenia, BD, or MDD, with 2 individuals overlapping) were randomly selected for review of the relative timing of the trauma and the index diagnosis (eFigure 1 and eTable 6 in Supplement 1).

### Mental Health Diagnoses

Mental health diagnoses were determined by aggregating related *International Classification of Diseases, Ninth Revision* (*ICD-9*) and *Tenth Revision* (*ICD-10*) diagnosis codes into phecodes using the R PheWAS package^42,43,44^ and requiring 2 component codes for each diagnosis (eMethods in Supplement 1). Across sites, a total of 711 individuals with schizophrenia, 3820 with BD, and 12 840 with MDD diagnoses were identified.

### PGSs

Details on genotyping, quality control, and determination of genetic similarity can be found in the eMethods in Supplement 1. In this study, we adopted recently reported guidelines on population descriptors for genomics research published by the National Academies of Sciences, Engineering, and Medicine (NASEM).^45^ PGSs for schizophrenia, BD, and MDD were generated for 84 955 genotyped individuals with high genetic similarity to the 1000 Genomes Project^46^ (1KG) CEU (Northern European from Utah) reference population (VUMC) or the combined European-ancestry superpopulation (MGB) using PRS-CS-auto,^47^ summary statistics from published GWAS,^48,49,50^ and a linkage disequilibrium reference panel constructed from 503 1KG European-ancestry individuals. Scores for 11 047 individuals with high genetic similarity to the 1KG YRI (Yoruba in Ibadan, Nigeria) reference population in the VUMC biobank were generated using PRS-CSx-auto^51^ with the meta option, leveraging cross-population discovery GWAS and linkage disequilibrium panels constructed from both 1KG European-ancestry (n = 503) and African-ancestry (n = 661) individuals (eTable 7 in Supplement 1). In accordance with NASEM recommendations, we refer to these study populations as *1KG-EU-clustered* or *1KG-YRI-clustered* throughout this article.

### Statistical Analysis

Multivariable logistic regressions were conducted in R statistical software versions 4.0.2 (for MGB data) and 4.2.1 (for VUMC data) (R Foundation),^52^ with each mental health diagnosis as the dependent variable and covariates for EHR-median age (median age at visit across all diagnostic codes in an individual’s record) and sex at minimum. For genetic analyses, the first 3 principal components estimated from genetic data were included as covariates to control for population stratification. In the gene-by-environment interaction analysis, PGS, sexual trauma history, and a multiplicative gene-by-environment interaction term were included as independent variables. In the main PGS analyses, odds ratios (ORs) per SD unit increase in PGS were calculated. In supplementary analyses, PGSs were dichotomized at the 75th percentile, and the interaction was evaluated on the additive (using the delta method^53^) and multiplicative scales. Meta-analyses were performed with inverse-variance-weighted meta-analysis using the meta^54^ R package. Goodness-of-fit analyses were performed using the rms^55^ R package. Gene-by-environment meta-analysis *P* values were Bonferroni adjusted for the 3 conditions tested. Corrected *P* < .05 with 2-tailed testing was considered statistically significant.

### Sensitivity Analyses

Four sensitivity analyses were performed to assess the robustness of results on controlling for possible sources of confounding. The possible sources were comorbid mental health conditions, substance use disorder (SUD) diagnoses, housing instability, and the clinical setting of the disclosures (eTable 8 and eMethods in Supplement 1).

## Results

Across the VUMC and MGB biobanks, 96 002 individuals were included in analyses. Cross-site meta-analyses included 84 955 1KG-EU-clustered individuals, with 58 262 from VUMC and 26 693 from MGB. More than half of the patients were female (VUMC, 33 011 [56.7%]; MGB, 14 647 [54.9%]) (Table 1), and the median age at visit ranged from 10 to more than 89 years (median [range], VUMC: 56.8 [10.0 to >89] years; MGB: 58.0 [10.0 to >89] years). An additional 11 047 1KG-YRI-clustered individuals from VUMC were included in analyses (6961 [63.0%] female; median [range] age at visit, 44.6 [10.1 to >89] years). Across cohorts, compared with those with no disclosures, individuals with documented sexual trauma disclosures were younger, more often female, and had longer and denser EHRs (eTable 1 in Supplement 1). Across cohorts, disclosures of sexual trauma were associated with increased odds of schizophrenia, BD, and MDD, with ORs ranging from 8.83 (95% CI, 5.50-14.18) for schizophrenia in the VUMC 1KG-YRI-clustered cohort to 17.65 (95% CI, 12.77-24.40) for schizophrenia in the VUMC 1KG-EU-clustered cohort (Table 2; eTable 9 in Supplement 2).

**Table 2.** Main Associations Between Sexual Trauma History and Mental Health Diagnoses^a^

Phenotype and cohort	Total No.	No. with diagnosis	OR (95% CI)^a^	*P* value
Schizophrenia				
VUMC 1KG-EU-clustered	58 119	274	17.65 (12.77-24.40)	<.001
MGB 1KG-EU-clustered	26 576	255	11.58 (8.46-15.85)	<.001
VUMC 1KG-YRI-clustered	11 002	182	8.83 (5.50-14.18)	<.001
Bipolar disorder				
VUMC 1KG-EU-clustered	57 208	1966	13.95 (11.89-16.38)	<.001
MGB 1KG-EU-clustered	26 219	1449	12.34 (10.43-14.59)	<.001
VUMC 1KG-YRI-clustered	10 850	405	13.48 (9.90-18.36)	<.001
Major depressive disorder				
VUMC 1KG-EU-clustered	55 470	5965	10.81 (9.31-12.56)	<.001
MGB 1KG-EU-clustered	25 250	5849	11.60 (9.67-13.91)	<.001
VUMC 1KG-YRI-clustered	10 527	1026	11.64 (8.77-15.45)	<.001

Abbreviations: MGB, Mass General Brigham; OR, odds ratio; VUMC, Vanderbilt University Medical Center; 1KG-EU-clustered, 1000 Genomes Project European–clustered; 1KG-YRI-clustered, 1000 Genomes Project Yoruba in Ibadan, Nigeria–clustered.

^a^ All models are adjusted for electronic health record–median age (median age at visit across all diagnostic codes in an individual’s record) and electronic health record–recorded sex.

### PGS Analyses

**Figure 1.**
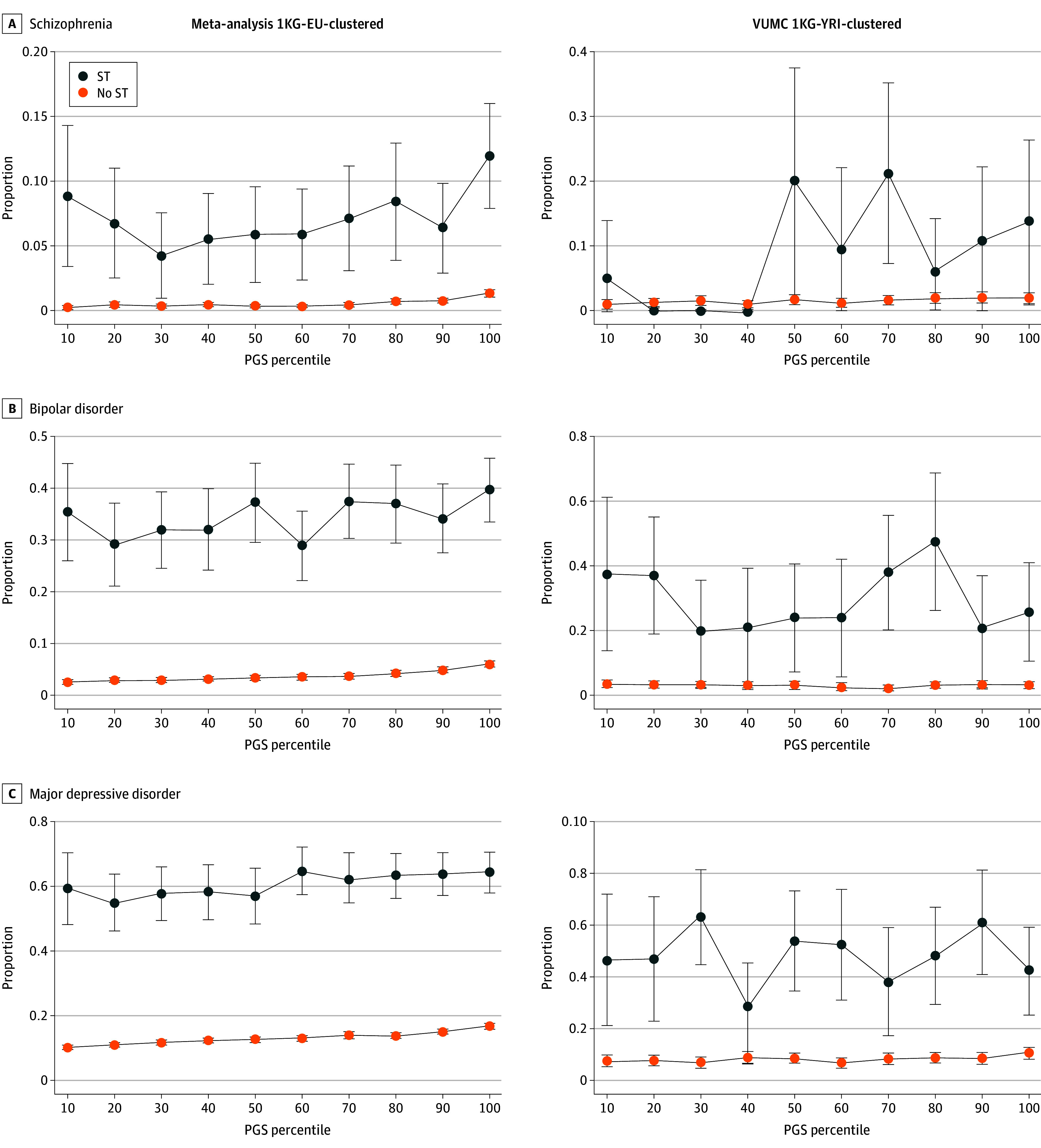
Case Proportions for Each Mental Health Diagnosis, Across Polygenic Score (PGS) Deciles and Stratified by Sexual Trauma (ST) History Cross-site pooled proportion estimates for the 1000 Genomes Project European (1KG-EU)–clustered meta-analysis study population, as well as proportion estimates for the Vanderbilt University Medical Center (VUMC) 1000 Genomes Project Yoruba in Ibadan, Nigeria (1KG-YRI)–clustered study population, are shown. Whiskers represent 95% CIs around prevalence estimates. Points with no 95% CIs indicate a prevalence estimate of 0 in those deciles.

**Figure 2.**
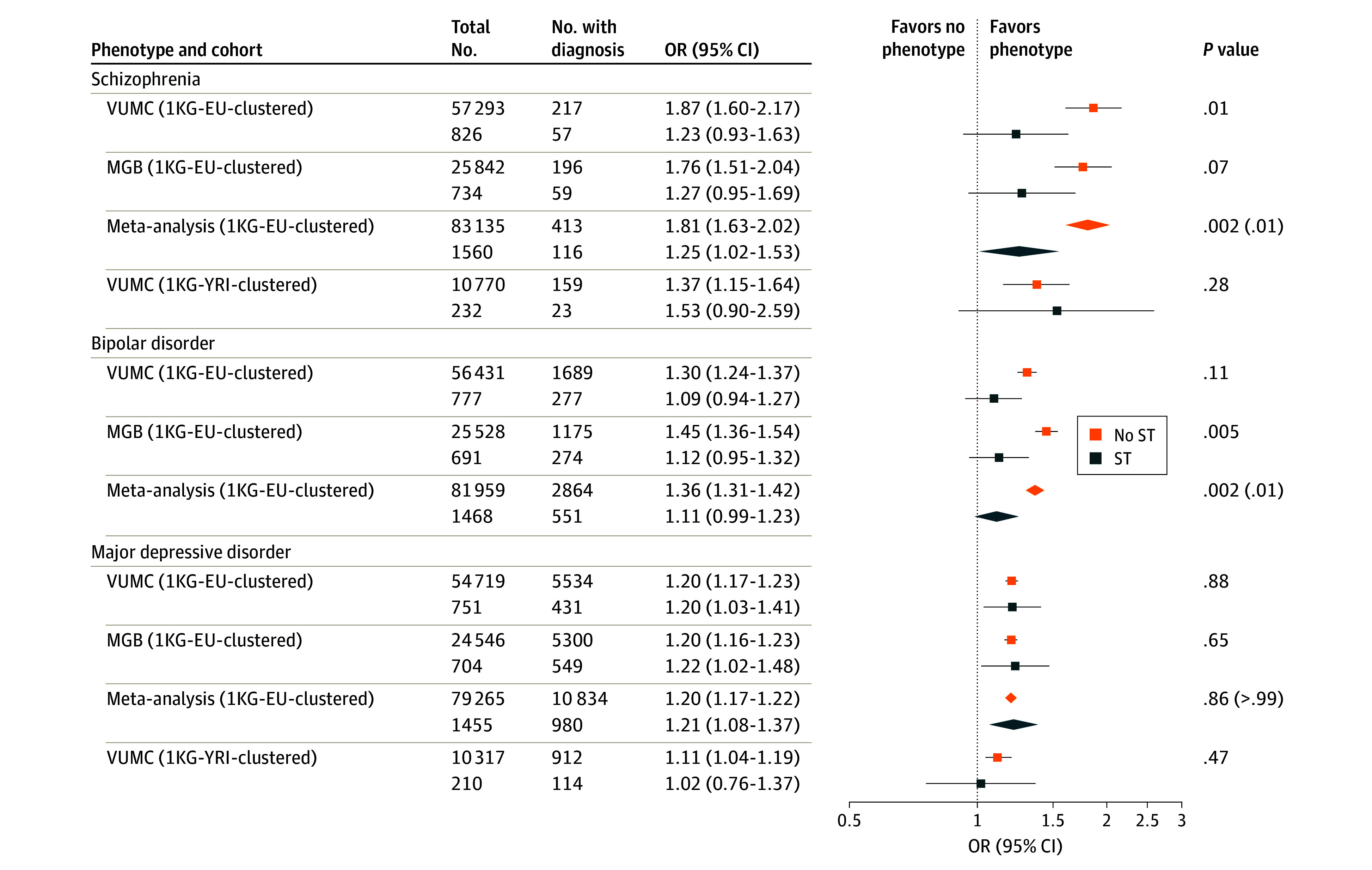
Site-Specific and Meta-Analysis Mental Health Odds Ratios (ORs) per SD Unit Increase in Polygenic Score, Stratified by Sexual Trauma (ST) History Multiplicative gene-by-environment interaction *P* values for each condition are shown, with Bonferroni-adjusted (n = 3) *P* values included for each meta-analysis. All models are adjusted for electronic health record–median age (median age at visit across all diagnostic codes in an individual’s record) and sex and the first 3 principal components estimated from genetic data. MGB indicates Mass General Brigham; VUMC, Vanderbilt University Medical Center; 1KG-EU-clustered, 1000 Genomes Project European–clustered; 1KG-YRI, 1000 Genomes Project Yoruba in Ibadan, Nigeria–clustered.

Stratified case proportion estimates across PGS deciles for each diagnosis are visualized in Figure 1. In the cross-site meta-analysis, as expected, among individuals without disclosures of sexual trauma, each diagnosis was associated with a linear increase in its respective PGS (OR per SD unit increase for schizophrenia, 1.81 [95% CI, 1.63-2.02]; BD, 1.36 [95% CI, 1.31-1.42]; MDD, 1.20 [95% CI, 1.17-1.22]) (Figure 1, Figure 2; eTable 10 in Supplement 2). In the VUMC 1KG-YRI-clustered population, this was the case for schizophrenia (OR, 1.37 [95% CI, 1.15-1.64]) and MDD (OR, 1.11 [95% CI, 1.04-1.19]) but not BD (OR, 1.01 [95% CI, 0.90-1.14]) (Figure 1, Figure 2; eTable 10 in Supplement 2), likely due to low sample sizes in the discovery GWAS and the genetic distance from the PGS training data. Consequently, analyses of BD were not carried forward in this study population.

When restricting to individuals with sexual trauma disclosures, the meta-analysis associations were attenuated in schizophrenia (OR, 1.25 [95% CI, 1.02-1.53]) and BD (OR, 1.11 [95% CI, 0.99-1.23]) but unchanged in MDD (OR, 1.21 [95% CI, 1.08-1.37]) (Figure 1 and Figure 2). This attenuation was confirmed by a significant multiplicative gene-by-environment interaction in schizophrenia (interaction OR, 0.70 [95% CI, 0.56-0.88]; Bonferroni-adjusted *P* = .006) and BD (interaction OR, 0.83 [95% CI, 0.74-0.94]; Bonferroni-adjusted *P* = .006) but not in MDD (interaction OR, 1.01 [95% CI, 0.90-1.14]) (Figure 2; eTable 10 in Supplement 2). The interaction analysis was repeated using PGSs dichotomized at the 75th percentile with similar multiplicative interaction results, but no interaction on the additive scale was detected (eFigure 2 and eTable 11 in Supplement 1). No significant interaction was observed in the 1KG-YRI-clustered population (Figure 2; eTable 10 in Supplement 2; eTable 11 in Supplement 1).

We used goodness-of-fit (pseudo-*R*^2^) analysis to evaluate the contributions of PGS, sexual trauma, and their interactions to mental health phenotypic variance.^56^ Across cohorts, PGS and sexual trauma contributed significantly to phenotypic variance when modeled independently or jointly, and joint models explained an additional 3.8% to 8.8% of the phenotypic variance beyond demographic variables (eFigure 3 in Supplement 1; eTable 12 in Supplement 2). While the gene-by-environment interaction reached statistical significance in schizophrenia (VUMC 1KG-EU-clustered) and BD (MGB 1KG-EU-clustered), the interaction term only increased overall phenotypic variance explained in these cohorts by a minuscule amount (<0.2%).

### Sensitivity Analyses

Due to the overlapping symptoms among psychiatric conditions, we conducted sensitivity analyses in which, in the regression for each condition, we adjusted for the presence of each of the other 2 conditions. While the associations between sexual trauma and mental health diagnoses were attenuated and the additional variance explained by the joint model beyond covariates alone was reduced (1.2%-3.3%), the results were otherwise largely unchanged (eTable 13, eFigure 4, and eFigure 5 in Supplement 1; eTable 9 and eTable 10 in Supplement 2).

Gene-environment correlation is known to influence gene-by-environment interaction studies^57^ and has been reported in polygenic analyses of childhood trauma,^22,34,58^ intimate partner violence,^59^ and other stressful life events.^60^ In our dataset, we observed modest positive associations between sexual trauma and PGS, with ORs ranging from 1.21 (95% CI, 1.13-1.31) to 1.38 (95% CI, 1.28-1.49) (eTable 14 in Supplement 1; eTable 10 in Supplement 2). Under the assumption that sexual trauma is a mediator between mental health PGS and diagnoses, the estimation of trauma-stratified associations of PGS with diagnosis risk may induce collider bias due to unmeasured confounding between sexual trauma and diagnosis.^61^ We conducted simulation analyses using the simDAG R package^62^ (eMethods in Supplement 1) to model this bias and found that, while gene-environment correlation alone is not expected to affect the interaction estimates (eFigure 6A in Supplement 1), in the presence of unadjusted confounders between sexual trauma and diagnosis, a gene-by-environment interaction may be induced (eFigure 6B and C in Supplement 1). We therefore conducted sensitivity analyses adjusting for additional potential confounders between sexual trauma and mental health diagnoses.

One potential source of confounding of the associations between sexual trauma and psychiatric conditions is the clinical setting of the disclosures, in particular if patients are more likely to be screened for trauma history in a psychiatric setting. We found that 32% of individuals disclosing a history of sexual trauma had evidence of a psychiatric visit on the date of the earliest disclosure (eMethods, eTable 15, and eTable 16 in Supplement 1). To mitigate this potential bias, we repeated the meta-analyses after excluding these individuals. We observed that sexual trauma and each diagnosis remained associated, that the interactions in schizophrenia and BD remained significant, and that sexual trauma and PGS each remained significant contributors to mental health phenotypic variance, with the additional variance explained by the joint model decreasing to 2.3% to 7.8% (eTable 17, eFigure 7, and eFigure 8 in Supplement 1; eTables 9, 10, and 12 in Supplement 2).

Additional potential sources of confounding of the associations between sexual trauma and psychiatric diagnoses that can be evaluated using EHRs are SUDs and social determinants of health, such as housing instability. These factors can increase vulnerability to sexual trauma and are associated with poor mental health.^63,64,65,66,67^ We therefore repeated our analyses after including SUD diagnoses and diagnosis codes relating to housing instability as covariates in separate sensitivity analyses (eTables 18 and 19 and eFigures 9-12 in Supplement 1; eTables 9, 10, and 12 in Supplement 2). In both analyses, sexual trauma and each diagnosis remained associated, and sexual trauma and PGS remained significant contributors to mental health phenotypic variance across cohorts (eTable 18, eTable 19, eFigure 10, and eFigure 12 in Supplement 1), with additional variance explained by the joint model beyond demographic variables decreasing to 2.4% to 5.8% (SUD analysis) or 2.9% to 6.8% (housing instability analysis). The gene-by-environment interaction in schizophrenia and BD remained significant across both analyses (eFigure 9 and eFigure 11 in Supplement 1).

## Discussion

Our study revealed several key findings. First, we identified an association between sexual trauma and mental health diagnoses across institutions, which was robust to confounding by comorbid psychiatric conditions, SUD diagnoses, and documented housing instability. Although disclosures of sexual trauma were overrepresented in psychiatric clinical settings due to increased screening, we showed that these associations persisted when restricting to disclosures that were not documented in a psychiatric setting.

Second, PGS and sexual trauma were found to be independent and joint risk factors for schizophrenia, BD, and MDD across all but 1 cohort. Specifically, the BD PGS was not significantly associated with BD diagnosis in the 1KG-YRI-clustered sample, likely reflecting differences in linkage disequilibrium patterns between the population used to train the BD PGS and the study population. While the joint contributions of sexual trauma and PGS accounted for less phenotypic variance after adjusting for comorbid conditions, clinical setting, SUD diagnosis, and housing instability, these factors remained significantly associated with each diagnosis both independently and in joint models across all analyses.

Third, in our cross-site meta-analysis, we observed gene-by-environment interactions between PGS and sexual trauma in schizophrenia and BD, but not MDD, in which the association between each PGS and its respective diagnosis was greater in patients without disclosures of sexual trauma. One interpretation of these findings is that the association between sexual trauma and mental health outcomes is sufficiently impactful to outweigh additional phenotypic variance explained by PGS, similar to previous observations of reduced heritability of traits in the context of social or environmental stressors.^68,69^ This study relies on disclosures documented in clinical notes and thus may disproportionately identify severely affected help-seeking individuals who were experiencing health consequences of their trauma, as evidenced by the increased indicators of health care utilization in trauma-disclosing individuals (eTable 1 in Supplement 1). Under this assumption, the experience of trauma may have been sufficient to predispose trauma-reporting individuals to developing psychiatric illness, with relatively less contribution from underlying genetic predisposition—at least that captured by PGSs derived from existing GWASs.

Notably, our meta-analysis identified significant gene-by-environment interactions in schizophrenia and BD but not in MDD. While our analyses of MDD have increased sample size due to the higher prevalence of the diagnosis relative to schizophrenia and BD, heritability estimates for MDD, as well as the magnitude of risk conferred by its PGS, are lower than for the other conditions. Therefore, it may be more difficult to detect an attenuation in the association of the PGS when the baseline association is relatively modest.

### Limitations

The results of this study should be considered in light of several limitations. First, our analyses in individuals with majority African ancestries are limited in sample size, and the BD PGS is not associated with the BD diagnosis in this study population, likely due to limited genetic similarity to the discovery GWAS populations. This limitation emphasizes the need for increased population diversity in existing GWAS efforts. However, despite being underpowered to detect an interaction, we established that sexual trauma and polygenic liability remained independently and jointly associated with schizophrenia and MDD in this study population.

Second, EHR-derived datasets are inherently subject to diagnostic imprecision. We took steps to mitigate potential effects of phenotypic overlap on our analyses by performing sensitivity analyses in which we adjusted for the presence of comorbid mental health diagnoses and available data on social determinants of health. Further, while our sexual trauma disclosure algorithm has relatively high precision, we believe sensitivity remains low as it only identified approximately 2% of patients across the 2 biobanks as having a disclosure, in contrast to the much higher prevalence estimates for sexual trauma in the underlying population.^1,2^ This discrepancy is likely due to underreporting of trauma in health care settings and algorithmic sensitivity. Importantly, the findings of this study should be interpreted in light of this limitation and may not generalize to the broader population of individuals with a history of sexual trauma. Rather, given the cross-site replicability of the results, our findings can be considered generalizable to individuals with a history of sexual trauma whose experiences were recorded in their medical records. This group may represent a subpopulation of help-seeking individuals who have experienced sexual trauma.

Third, gene-environment correlation can impact studies of gene-by-environment interaction, and we indeed observed correlation between sexual trauma disclosures and mental health PGS in our study, which could arise due to passive, evocative, or active mechanisms.^57,58^ Our observation that the majority of documented sexual trauma occurred during childhood may point to a passive mechanism in which parental genetic predisposition to psychiatric conditions influences the family environment. Our simulation results demonstrated that increasing gene-environment correlation did not directly affect the gene-by-environment interaction results, but that the presence of unmeasured confounding between sexual trauma and psychiatric outcomes can bias the interaction estimates proportional to increasing gene-environment correlation. We conducted several sensitivity analyses to mitigate this potential confounding but recognize that additional unmeasured confounders remain unaccounted for in our analyses. We also acknowledge that simulation studies are useful tools but are inherently limited in their ability to accurately represent real-world scenarios.

Fourth, given that sexual trauma disclosures in clinical notes can refer to experiences from one’s remote past, it is difficult to algorithmically determine the precise time in the patient’s life at which the trauma occurred. Extrapolating from a manual review of 50 records (eTable 5 in Supplement 1), we found that due to the majority (74%) of reported traumas likely occurring during childhood, it is likely that most reported traumas occurred before the onset of the mental health condition. We further verified this assumption by performing an additional manual record review of individuals with both trauma disclosures and each of the psychiatric diagnoses and found that across diagnoses, in 83% to 95% of manually reviewed records with sufficient documentation to make a determination, the contextual information in the notes indicated that the reported trauma likely predated the diagnosis (eTable 6 in Supplement 1).

## Conclusions

Taken together, our findings suggest that sexual trauma and mental health PGSs, while intercorrelated, are important risk factors for schizophrenia, BD, and MDD diagnoses and that schizophrenia and BD PGSs generated using existing methods may be less predictive in the context of sexual trauma history. As PGSs are being considered for use in clinical settings, these findings demonstrate the importance of expanding clinical screening efforts for sexual trauma and other environmental risk factors that may impact the clinical interpretation of the PGSs. Future work developing algorithms to identify a broad set of environmental exposures is warranted to enable the comprehensive examination of gene-by-environment interactions in real-world clinical datasets for the implementation of precision medicine and psychiatry.

## Notes

Supplement 1.
eMethods
**eTable 1. **Sample Characteristics, Stratified by Sexual Trauma Disclosure Status **eTable 2. **Inclusion Phrases Used in Sexual Trauma Disclosure Detection Algorithm **eTable 3. **Exclusion Phrases Used in Sexual Trauma Disclosure Detection Algorithm **eTable 4. **Sexual Trauma Disclosure Algorithm Performance Metrics **eTable 5. **Timing of Sexual Trauma Documented in the VUMC EHR **eTable 6. **Timing of Documented Sexual Trauma in Relation to Mental Health Diagnoses in the VUMC EHR **eFigure 1. **Data Entry Tool Used for Chart Review Determining the Timing of the Reported Trauma in Relation to the Mental Health Diagnosis **eTable 7. **Genome-Wide Association Studies Used to Generate Polygenic Scores **eTable 8. **Diagnosis Codes Relating to Housing Instability **eFigure 2. **Site-Specific and Meta-Analysis Odds Ratios for Association Between Each Mental Health Condition and Its Respective Dichotomized Polygenic Score, Stratified Into Those With or Without Sexual Trauma Disclosures **eTable 11. **GxE Interaction Results on the Additive Scale With Polygenic Score Dichotomized at the 75th Percentile Within Each Study Population **eFigure 3. **Goodness of Fit (Pseudo-*R*^2^) Statistics for Each Regression Model in the Main GxE Interaction Analysis **eTable 13. **Main Effect Associations Between Sexual Trauma History and Mental Health Diagnoses After Adjusting for Comorbid Mental Health Diagnoses **eFigure 4. **GxE Interaction Results From the Comorbid Mental Health Condition Sensitivity Analysis **eFigure 5. **Goodness of Fit (Pseudo-*R*^2^) Statistics for Each Regression Model After Adjusting for Comorbid Mental Health Conditions **eTable 14. **Associations Between Sexual Trauma History and Polygenic Scores **eFigure 6. **Results of Simulation Analyses **eTable 15. **Procedure Codes Used for Identifying Psychiatry Visits **eTable 16. **Results of Mapping Sexual Trauma Disclosure Notes to Clinical Settings **eTable 17. **Main Effect Associations Between Sexual Trauma History and Mental Health Diagnoses After Removing Sexual Trauma Cases Whose Initial Disclosures Were Reported in a Psychiatry Setting (Clinical Setting Sensitivity Analysis) **eFigure 7. **GxE Interaction Results From the Clinical Setting Sensitivity Analysis **eFigure 8. **Goodness of Fit (Pseudo-*R*^2^) Statistics for Each Regression Model in the Clinical Setting Sensitivity Analysis **eTable 18. **Main Effect Associations Between Sexual Trauma History and Mental Health Diagnoses After Adjusting for Substance Use Disorder Diagnoses **eTable 19. **Main Effect Associations Between Sexual Trauma History and Mental Health Diagnoses After Adjusting for Billing Codes Relating to Housing Instability **eFigure 9. **GxE Interaction Results From the Substance Use Disorder Sensitivity Analysis **eFigure 10. **Goodness of Fit (Pseudo-*R*^2^) Statistics for Each Regression Model in the Substance Use Disorder Sensitivity Analysis **eFigure 11. **GxE Interaction Results From the Housing Instability Sensitivity Analysis **eFigure 12. **Goodness of Fit (Pseudo-*R*^2^) Statistics for Each Regression Model in the Housing Instability Sensitivity Analysis
eReferences

Supplement 2. **eTable 9.** Full Sexual Trauma-Diagnosis Association (Main Effect) Results for Main Analysis and Sensitivity Analyses **eTable 10.** Full GxE Regression Results for Main Analysis and Sensitivity Analyses **eTable 12.** Goodness of Fit (Pseduo-*R*^2^) Statistics for Main Analysis and Sensitivity Analyses

Supplement 3. Data Sharing Statement
