# Supplementary material for: Sexual Trauma, Polygenic Scores, and Mental Health Diagnoses and Outcomes
Source: JAMA Psychiatry. 2024 Oct 30;82(1):75–84. doi: 10.1001/jamapsychiatry.2024.3426 (PMC11581726; doi:10.1001/jamapsychiatry.2024.3426)
Supplement: Supplement 1. — eMethods eTable 1. Sample Characteristics, Stratified by Sexual Trauma Disclosure Status eTable 2. Inclusion Phrases Used in Sexual Trauma Disclosure Detection Algorithm eTable 3. Exclusion Phrases Used in Sexual Trauma Disclosure Detection Algorithm eTable 4. Sexual Trauma Disclosure Algorithm Performance Metrics eTable 5. Timing of Sexual Trauma Documented in the VUMC EHR eTable 6. Timing of Documented Sexual Trauma in Relation to Mental Health Diagnoses in the VUMC EHR eFigure 1. Data Entry Tool Used for Chart Review Determining the Timing of the Reported Trauma in Relation to the Mental Health Diagnosis eTable 7. Genome-Wide Association Studies Used to Generate Polygenic Scores eTable 8. Diagnosis Codes Relating to Housing Instability eFigure 2. Site-Specific and Meta-Analysis Odds Ratios for Association Between Each Mental Health Condition and Its Respective Dichotomized Polygenic Score, Stratified Into Those With or Without Sexual Trauma Disclosures eTable 11. GxE Interaction Results on the Additive Scale With Polygenic Score Dichotomized at the 75th Percentile Within Each Study Population eFigure 3. Goodness of Fit (Pseudo-R2) Statistics for Each Regression Model in the Main GxE Interaction Analysis eTable 13. Main Effect Associations Between Sexual Trauma History and Mental Health Diagnoses After Adjusting for Comorbid Mental Health Diagnoses eFigure 4. GxE Interaction Results From the Comorbid Mental Health Condition Sensitivity Analysis eFigure 5. Goodness of Fit (Pseudo-R2) Statistics for Each Regression Model After Adjusting for Comorbid Mental Health Conditions eTable 14. Associations Between Sexual Trauma History and Polygenic Scores eFigure 6. Results of Simulation Analyses eTable 15. Procedure Codes Used for Identifying Psychiatry Visits eTable 16. Results of Mapping Sexual Trauma Disclosure Notes to Clinical Settings eTable 17. Main Effect Associations Between Sexual Trauma History and Mental Health Diagnoses After Removing Sexual Trauma Cases Whose [file jamapsychiatry-e243426-s001.pdf]

Supplemental Online Content

Lake AM, Zhou Y, Wang B, et al. Sexual trauma, polygenic scores, and mental health diagnoses and outcomes. *JAMA Psychiatry*. Published online October 30, 2024. doi:10.1001/jamapsychiatry.2024.3426

eMethods
eTable 1. Sample Characteristics, Stratified by Sexual Trauma Disclosure Status
eTable 2. Inclusion Phrases Used in Sexual Trauma Disclosure Detection Algorithm
eTable 3. Exclusion Phrases Used in Sexual Trauma Disclosure Detection Algorithm
eTable 4. Sexual Trauma Disclosure Algorithm Performance Metrics
eTable 5. Timing of Sexual Trauma Documented in the VUMC EHR
eTable 6. Timing of Documented Sexual Trauma in Relation to Mental Health Diagnoses in the VUMC EHR
eFigure 1. Data Entry Tool Used for Chart Review Determining the Timing of the Reported Trauma in Relation to the Mental Health Diagnosis
eTable 7. Genome-Wide Association Studies Used to Generate Polygenic Scores
eTable 8. Diagnosis Codes Relating to Housing Instability
eFigure 2. Site-Specific and Meta-Analysis Odds Ratios for Association Between Each Mental Health Condition and Its Respective Dichotomized Polygenic Score, Stratified Into Those With or Without Sexual Trauma Disclosures
eTable 11. GxE Interaction Results on the Additive Scale With Polygenic Score Dichotomized at the 75th Percentile Within Each Study Population
eFigure 3. Goodness of Fit (Pseudo-R^2) Statistics for Each Regression Model in the Main GxE Interaction Analysis
eTable 13. Main Effect Associations Between Sexual Trauma History and Mental Health Diagnoses After Adjusting for Comorbid Mental Health Diagnoses
eFigure 4. GxE Interaction Results From the Comorbid Mental Health Condition Sensitivity Analysis
eFigure 5. Goodness of Fit (Pseudo-R^2) Statistics for Each Regression Model After Adjusting for Comorbid Mental Health Conditions
eTable 14. Associations Between Sexual Trauma History and Polygenic Scores
eFigure 6. Results of Simulation Analyses
eTable 15. Procedure Codes Used for Identifying Psychiatry Visits
eTable 16. Results of Mapping Sexual Trauma Disclosure Notes to Clinical Settings
eTable 17. Main Effect Associations Between Sexual Trauma History and Mental Health Diagnoses After Removing Sexual Trauma Cases Whose Initial Disclosures Were Reported in a Psychiatry Setting (Clinical Setting Sensitivity Analysis)
eFigure 7. GxE Interaction Results From the Clinical Setting Sensitivity Analysis
eFigure 8. Goodness of Fit (Pseudo-R^2) Statistics for Each Regression Model in the Clinical Setting Sensitivity Analysis

© 2024 Lake AM et al. *JAMA Psychiatry.*

eTable 18. Main Effect Associations Between Sexual Trauma History and Mental Health Diagnoses After Adjusting for Substance Use Disorder Diagnoses
eTable 19. Main Effect Associations Between Sexual Trauma History and Mental Health Diagnoses After Adjusting for Billing Codes Relating to Housing Instability
eFigure 9. GxE Interaction Results From the Substance Use Disorder Sensitivity Analysis
eFigure 10. Goodness of Fit (Pseudo-R^2) Statistics for Each Regression Model in the Substance Use Disorder Sensitivity Analysis
eFigure 11. GxE Interaction Results From the Housing Instability Sensitivity Analysis
eFigure 12. Goodness of Fit (Pseudo-R^2) Statistics for Each Regression Model in the Housing Instability Sensitivity Analysis
eReferences

This supplemental material has been provided by the authors to give readers additional information about their work.

eMethods

Sample and genotype information

Vanderbilt University Medical Center (VUMC)
VUMC is a large tertiary care and academic medical center Nashville, Tennessee with a catchment area extending from southern Kentucky to northern Alabama. This study used the Synthetic Derivative (SD), a de-identified copy of the VUMC EHR. Data elements used in this study include structured data (diagnosis codes, procedure codes, demographics) and unstructured data (de-identified clinical notes). Genotyping data used in this study was obtained from BioVU, the biorepository at VUMC established in 2007, which banks leftover blood samples from routine clinical care[1]. Each DNA sample in BioVU is linked to the participant's de-identified EHR. The BioVU consent form is provided to patients in outpatient clinic environments at VUMC. All BioVU participants provided informed consent for inclusion in the biobank for broad-based research (IRB #041020).

The SD de-identification process involves the shifting of all dates backwards by up to 364 days and removing all HIPAA identifiers including name, all elements of the patient's address except state and the first 3 digits of the ZIP code, telephone number, fax number, email address, Social Security Number, medical record number (MRN), health plan beneficiary number, account number, certificate or license number, vehicle and device identifiers, web URL, IP address, finger or voice print, and photographic images. The de-identification process also removes all patient data, except for death data, recorded after an individual has reached 90 years of age.

The de-identification of SD records is achieved through the application of several methods including use of a commercial electronic program, which was applied and assessed for acceptable effectiveness in scrubbing identifiers. However, complete anonymization of granular healthcare data is not possible. To ensure confidentiality and appropriate use of the SD, all relevant key personnel enter into data use agreement, which prohibits any use of the data not described IRB protocol, including the re-identification of the SD / BioVU records. This study was approved by the VUMC Institutional Review Board and received an informed consent waiver and "non-human subjects" determination due to the use of deidentified medical record data (IRB #212285).

Genotyping for this study was performed using the Illumina Multi-Ethnic Genotyping Array ($MEGA^{EX}$) and quality controlled using standard procedures as previously described[2]. Genetic ancestry determination was conducted using principal component analysis (PCA) in FlashPCA 2.0[3]. Ancestry cluster boundaries were based on proportions from the center of mass of one reference population to another. The centers of mass (average PC value on each axis) for the Utah residents with Northern and Western European ancestry (CEU), Yoruba in Ibadan, Nigeria (YRI), and Han Chinese in Beijing, China (CHB) 1000 Genomes Project[4] (1KG) reference populations were determined. For the European-ancestry set, referred to as "1KG-EU-clustered" in this manuscript, inclusion boundaries around the CEU cluster in the PC1 vs PC2 space was defined as <30% along CEU-YRI axis and <40% along the CEU-CHB axis. The African-ancestry set, referred to as "1KG-YRI-clustered" in this manuscript, was defined as <70% of the YRI cluster along the YRI-CEU axis and <50% of the YRI cluster along the YRI-CHB axis. A total of 72,824 1KG-EU-clustered individuals and 15,283 1KG-YRI-clustered individuals were identified and retained for downstream analyses. Principal components for the control of population stratification were estimated using genetic data from these individuals. Imputation was performed using the Michigan Imputation Server[5] using the Haplotype Reference Consortium (HRC) reference panel, and related individuals were removed using an identity by descent (IBD) proportion filter of 0.2. A total of 58,262 1KG-EU-clustered and 11,047 1KG-YRI-clustered individuals remained after applying the IBD filter and additional cohort selection criteria for this study (age requirements, data floor), detailed in the **Methods** section of the main text.

Statistical analyses of VUMC data, as well as cross-site meta-analyses, were performed using R version 4.2.1[6].

Mass General Brigham (MGB)
MGB is a large healthcare system that provides clinical care to patients in the Greater Boston area and beyond. For this study, RPDRml, a de-identified version of the MGB EHR in which dates are shifted by a random number of days for each patient, was used to extract structured clinical data (diagnosis codes, procedure codes, demographics) for individuals participating in the MGB Biobank (formerly the Partners Biobank)[7] with an available DNA sample. Unstructured data (clinical notes) were linked to de-identified RPDRml and MGB Biobank subject IDs using a mapping file stored separate from the data in a secure password protected file accessible only to IRB approved study staff and the designated RPDRml "honest broker." The validation of the sexual trauma phenotyping algorithm via manual chart review required access to identifiable medical records. Only approved investigators or study staff had access to personal identifiers for this chart review. All data were deidentified prior to statistical analysis. The MGB IRB (protocol #2018P002642) approved this study and granted an informed consent waiver due to the use of retrospective medical record data with no patient interaction. All participants provided written informed consent to participate in the MGB Biobank for broad-based research (IRB #2009P002312).

Genotyping of 47,321 individuals in the MGB Biobank was performed using the Illumina Global Screening Array, and standard quality control procedures were performed as described (https://github.com/getian107/MGBB-QC). Imputation to the HRC reference panel was completed using the Michigan Imputation Server[5]. PCA was performed against the 1000 Genomes reference panel (including European, Admixed American, African, East Asian and South Asian super-populations), and ancestry group assignments were made based on random forest with a prediction probability more than 0.8. A total of 33,067 participants of primarily European ancestries based on the 1000 Genomes CEU, Tuscans from Italy (TSI), British in England and Scotland (GBR), Finnish in Finland (FIN), and Iberian populations in Spain (IBS) reference populations were retained for analysis and referred to as "1KG-EU-clustered" in this manuscript. PCs estimated from this population were used as covariates in polygenic score regression analyses to control for population stratification. Clinical data were obtained using RPDRml, a deidentified version of the MGB EHR. Patients included met a minimum data floor criteria of at least 3 visits after 2005, each more than 30 days apart, with at least one clinical note of any kind. After applying additional cohort selection criteria for this study (age requirements, data floor, see **Methods** in the main text), 26,693 individuals remained and were retained for analysis.

Statistical analyses of MGB data were performed using R version 4.0.2[6].

Polygenic scoring
Polygenic scores (PGS) for schizophrenia, bipolar disorder (BD), and major depressive disorder (MDD) were generated using summary statistics from previously published discovery genome-wide association study (GWAS). Polygenic scores were generated using PRS-CS-auto[8] or PRS-CSx-auto[9]. At MGB, all variants in each set of discovery GWAS summary statistics were included. At VUMC, only variants with minor allele frequency >1% and imputation information score of >0.3 (when this field was available) in the discovery population were included. A full list of studies and corresponding variant counts can be found in **eTable 7**.

Discovery GWAS summary statistics with a European ancestry label were applied using PRS-CS-auto[8] to 58,262 genotyped individuals at VUMC and 26,693 genotyped individuals at MGB given the target samples' genetic similarity to the 1KG CEU (VUMC) or combined CEU, TSI, GBR, FIN, and IBS (MGB). A linkage disequilibrium (LD) reference panel constructed from 503 1KG European-ancestry individuals was used. We refer to this population as "1KG-EU-clustered" in accordance with the National Academies of Sciences, Engineering, and Medicine guidelines for population descriptors[10].

Polygenic scores for an additional 11,047 individuals with high genetic similarity to the 1KG-YRI population at VUMC (1KG-YRI-clustered) were generated using PRS-CSx-auto[9] with the "meta" option, leveraging cross-population discovery GWAS (**eTable 7**). Here, LD reference panels constructed from both 1KG European-ancestry (N=503) and African-ancestry (N=661) individuals were used.

© 2024 Lake AM et al. *JAMA Psychiatry.*

To our knowledge, no genetic samples from VUMC were included in any of the discovery GWAS. It is possible that a small number of MGB samples were included in the bipolar disorder[11] and depression[12] discovery GWAS, but these individuals could not be identified.

Sexual trauma disclosure algorithm

Disclosures of sexual trauma were identified by mining text from clinical notes and using regular expressions to identify matches to key words and phrases, listed in **eTable 2** and **eTable 3.** Individuals with ≥1 clinical note containing ≥1 of the specified inclusion strings were identified as potential cases. Among potential cases, individuals with at least one clinical note containing at least one of the exclusion strings were excluded. At VUMC, the algorithm was applied to all available note types with the exception of radiology reports and family history. At MGB, the algorithm was applied to discharge summaries, progress notes, and visit notes. The algorithm has been deposited in PheKB at accession #1713 (https://phekb.org/phenotype/1713).

For the primary algorithm validation chart review, manual review of 50 randomly selected charts was performed by medical student AML (VUMC) and clinical psychologist KWC (MGB). The context surrounding each disclosure was reviewed. A disclosure was identified as a "false positive" if the disclosure did not indicate a patient report of a sexual assault. The most common examples of false positive reporting included disclosures that pertained to someone other than the patient (e.g., "her sister reported a rape") or if it was a negation or denial of sexual trauma (e.g., "he denied past trauma or history of sexual assault"). At MGB, after review of the first 25 of 50 charts, it was noted that some false positive cases contained negations including the word "any" (e.g., "she denied any history of sexual abuse"), and the algorithm exclusion phrases were updated accordingly (**eTable 3**). This updated algorithm was then used in all analyses at both VUMC and MGB.

The VUMC chart review aimed at determining whether the reported trauma occurred during childhood or adulthood (**eTable 5**) was performed by AML by examining the context surrounding the mentions of sexual trauma keywords in the clinical notes of the 46 "true positive" individuals from the primary chart review. Trauma episodes were classified as likely occurring in childhood if the timing was explicitly stated in the notes (e.g., "reported a history of sexual abuse during childhood") or if the timing was implied by the surrounding context (e.g., mention of "sexually abused by father" with age information redacted in combination with a mention of childhood trauma elsewhere in the clinical notes). Episodes were similarly classified as likely occurring during adulthood. Of the 46 "true positive" individuals, disclosures from 8 individuals could not be classified due to missing information (**eTable 5**).

The VUMC chart review aimed at determining the timing of the reported trauma in relation to the mental health diagnosis (**eTable 6**) was performed by AML and KVA. Longitudinal clinical notes and billing codes for each mental health diagnosis were used in the review. An initial set of 9 randomly sampled charts with both a sexual trauma disclosure and a mental health diagnosis (3 individuals each with schizophrenia, BD, or MDD, respectively) were reviewed by both reviewers with inter-rater concordance in 7 of 9 charts (78%). An additional 11 individuals per reviewer per diagnosis were sampled and reviewed independently (22 additional individuals per diagnosis) for a total of 73 individuals reviewed (with two individuals overlapping between conditions). The two reviewers collaborated and came to a joint consensus for charts in which the timing of assault and mental health diagnosis was not clearly indicated. The data entry tool shown in **eFigure 1** was used for review.

Mental health diagnoses

Diagnostic labels of each mental health condition were assigned by aggregating ICD-9 and ICD-10 billing codes into phecodes (schizophrenia, 295.1; BD, 296.1; MDD, 296.22) using the R PheWAS package[13–15]. At VUMC, to be labeled as a case, an individual must have documentation of any component billing code on at least two distinct dates. At MGB, to be labeled as a case, an individual must have documentation of any component billing code at least twice (regardless of date). At both sites, individuals with only one component code documented for the primary diagnosis being analyzed were excluded from the analysis to account for potential diagnostic inaccuracy. No exclusions based on comorbid diagnoses were applied to cases or controls.

Sensitivity analyses adjusting for comorbid mental health diagnoses, substance use disorders, and housing instability

For sensitivity analyses controlling for the presence of comorbid mental health conditions, individuals with only one component code of the comorbid condition (e.g., a comorbid code for MDD in the schizophrenia analysis) were not excluded from the analysis as above but rather were retained as controls for the comorbid diagnosis.

For the sensitivity analyses controlling for the presence of substance use disorder (SUD), phecodes for “substance addiction and disorders” (316), “alcohol-related disorders” (317), “alcoholism” (317.1), “alcoholic liver damage” (317.11), and “tobacco use disorder” (318) were combined into a single SUD phenotype. Individuals with documentation of at least two component codes (VUMC, on ≥2 distinct dates; MGB, regardless of date) for any SUD phecode were labeled as cases, and all others were labeled as controls in the analysis.

For the housing instability sensitivity analyses, individuals with ≥1 relevant ICD-9 or -10 billing code (listed in **eTable 8**) were labeled as cases, and all others were labeled as controls.

Clinical settings of sexual trauma disclosures

To map sexual trauma disclosures to clinical settings, for each patient with a disclosure, the date of the earliest disclosure was identified (index disclosure). Index disclosures were then linked by date with procedure codes related to psychiatry or psychotherapy (listed in **eTable 15**). At VUMC only, index disclosures were additionally linked by date with clinical encounter records including a field for the clinical location of the encounter within the VUMC hospital system. Clinical locations were manually reviewed by two medical students (AML and JPS) and labeled by clinical specialty. Only 69% of index disclosures could be mapped to any clinical specialty, and 15% of index disclosures mapped to multiple specialties. At VUMC, an index disclosure was identified as psychiatry-associated if it mapped either to a psychiatry procedure code or to a psychiatry-labeled encounter. At MGB, an index disclosure was identified as psychiatry-associated if it mapped to a psychiatry procedure code. These data are summarized in **eTable 16.**

Simulation analyses

Simulated datasets of one million individuals each were generated using the simDAG R package[16]. Two sets of simulations were conducted in which the log-odds ratio between PGS and trauma varied between 0 (no association) and 3 (strong association) at intervals of 0.01, as illustrated in **eFigure 6A** and **eFigure 6C**. To evaluate possible bias due to unmeasured confounding of the trauma-mental health outcome association, the second set of simulations included an unmeasured confounder that was associated (log odds ratio=0.5) with sexual trauma and schizophrenia (**eFigure 6C**). The simulated effects for all other causal relationships were derived directly from the results of the main schizophrenia GxE regression analysis presented in **Figure 2.** Statistical analyses were conducted on each simulated dataset as described in the **Methods**.

© 2024 Lake AM et al. *JAMA Psychiatry.*

eTable 1. Sample characteristics, stratified by sexual trauma disclosure status

	VUMC 1KG-EU-clustered		MGB 1KG-EU-clustered		VUMC 1KG-YRI-clustered	
	ST N=844	No ST N=57,418	ST N=752	No ST N=25,941	ST N=238	No ST N=10,809
**EHR-recorded sex**						
Female	721 (85.4%)	32,290 (56.2%)	594 (79.0%)	14,053 (54.2%)	195 (81.9%)	6,766 (62.6%)
Male	123 (14.6%)	25,128 (43.8%)	158 (21.0%)	11,888 (45.8%)	43 (18.1%)	4,043 (37.4%)
**Median age[a] (years)**						
Mean (SD)	38.8 (16.8)	54.5 (18.2)	48.2 (14.9)	55.7 (16.3)	36.4 (15.8)	44.7 (18.4)
Median [Min, Max]	38.4 [10.3, 85.4]	57.0 [10.0, >89]	49.0 [16.0, 84.0]	58.0 [10.0, >89]	34.0 [10.9, 80.9]	44.9 [10.1, >89]
**Unique days[b]**						
Mean (SD)	159.3 (157.3)	84.7 (100.5)	381.9 (338.1)	168.7 (169.0)	142.8 (159.7)	77.6 (106.0)
Median [Min, Max]	107.0 [3.0, 1,390.0]	51.0 [3.0, 2,052.0]	281.0 [8.0, 2,330.0]	117.0 [3.0, 2,503.0]	89.5 [5.0, 1,142.0]	41.0 [3.0, 1,228.0]
**Record length (years)**						
Mean (SD)	15.9 (6.8)	11.3 (7.5)	17.1 (6.6)	14.8 (7.4)	15.5 (7.3)	11.1 (7.7)
Median [Min, Max]	16.4 [0.0, 32.6]	11.2 [0.0, 32.8]	18.9 [0.2, 24.4]	15.6 [0.1, 24.4]	16.2 [0.6, 32.5]	10.4 [0.0, 32.7]
**Schizophrenia**						
Yes	57 (6.8%)	217 (0.4%)	59 (7.8%)	196 (0.8%)	23 (9.7%)	159 (1.5%)
No	769 (91.1%)	57,076 (99.4%)	675 (89.8%)	25,646 (98.9%)	209 (87.8%)	10,611 (98.2%)
Excluded[c]	18 (2.1%)	125 (0.2%)	18 (2.4%)	99 (0.4%)	6 (2.5%)	39 (0.4%)
**Bipolar disorder**						
Yes	277 (32.8%)	1,689 (2.9%)	274 (36.4%)	1,175 (4.5%)	70 (29.4%)	335 (3.1%)
No	500 (59.2%)	54,742 (95.3%)	417 (55.5%)	24,353 (93.9%)	151 (63.4%)	10,294 (95.2%)
Excluded[c]	67 (7.9%)	987 (1.7%)	61 (8.1%)	413 (1.6%)	17 (7.1%)	180 (1.7%)

© 2024 Lake AM et al. *JAMA Psychiatry.*

**Major depressive disorder**						
Yes	431 (51.1%)	5,534 (9.6%)	549 (73.0%)	5,300 (20.4%)	114 (47.9%)	912 (8.4%)
No	320 (37.9%)	49,185 (85.7%)	155 (20.6%)	19,246 (74.2%)	96 (40.3%)	9,405 (87.0%)
Excluded[c]	93 (11.0%)	2,699 (4.7%)	48 (6.4%)	1,395 (5.4%)	28 (11.8%)	492 (4.6%)
**Substance use disorders**						
Yes	377 (44.7%)	10,658 (18.6%)	462 (61.4%)	7,094 (27.3%)	106 (44.5%)	1,880 (17.4%)
No	467 (55.3%)	46,760 (81.4%)	290 (38.6%)	18,847 (72.7%)	132 (55.5%)	8,929 (82.6%)
**Inadequate housing**						
Yes	62 (7.3%)	1,214 (2.1%)	110 (14.6%)	409 (1.6%)	21 (8.8%)	200 (1.9%)
No	782 (92.7%)	56,204 (97.9%)	642 (85.4%)	25,532 (98.4%)	217 (91.2%)	10,609 (98.1%)
**ST disclosure in psychiatry setting[d]**						
Yes	273 (32.3%)	N/A	235 (31.3%)	N/A	70 (29.4%)	N/A
No	571 (67.7%)		517 (68.8%)		168 (70.6%)	

Abbreviations: VUMC, Vanderbilt University Medical Center; MGB, Mass General Brigham; CI, confidence interval; ST, sexual trauma.

[a] Median age refers to the median age at visit across all diagnostic codes in an individual's record.
[b] Unique days refers to the total number of distinct visit dates associated with diagnostic codes.
[c] For a given diagnosis, individuals with a single component code but not meeting the full case definition (≥2 distinct code-dates) are excluded from statistical analyses of that diagnosis.
[d] For individuals with ST disclosures, the earliest disclosure was identified and linked with procedure code and/or encounter information to determine whether the disclosure co-occurred with a psychiatry visit.

© 2024 Lake AM et al. *JAMA Psychiatry.*

eTable 2. Inclusion phrases used in sexual trauma disclosure detection algorithm

	Key phrase
1	(history of |hx of |h/o)sexual (assault|abuse)
2	(his |her)rape
3	sexual (assault|abuse) by
4	sexually (assaulted|abused) by
5	(reports|reported) a rape
6	was raped
7	sexually (abused|assaulted) (him|her)
8	secondary to (rape|sexual assault|sexual abuse)

eTable 3. Exclusion phrases used in sexual trauma disclosure detection algorithm

	Key phrase
1	no (history of |hx of |h/o)sexual (assault|abuse)
2	(denies|denied) (history of |hx of |h/o)sexual (assault|abuse)
3	(denies|denied) any (history of |hx of |h/o)sexual (assault|abuse)

eTable 4. Sexual trauma disclosure algorithm performance metrics

Site	Charts reviewed	True positive cases	Positive predictive value (95% CI)
VUMC	50	46	92.0% (79.9%, 97.4%)
MGB[a]	50	40	80.0% (65.9%, 89.5%)

Abbreviations: VUMC, Vanderbilt University Medical Center; MGB, Mass General Brigham; CI, confidence interval.

[a] At MGB, the first 25 of 50 manually reviewed charts used a version of the algorithm not containing exclusion phrase set 3 in **eTable 3** (see **eMethods**).

© 2024 Lake AM et al. *JAMA Psychiatry.*

eTable 5. Timing of sexual trauma documented in the VUMC EHR

Timing of trauma	N	% Total[a]	% TP, not undetermined[b]
Likely childhood trauma	28	56.0%	73.7%
Likely adult trauma	10	20.0%	26.3%
Undetermined	8	16.0%	
Total TP disclosures (not undetermined)	38	76.0%	100%
Total TP disclosures	46	92.0%	
Grand total	50	100%	

Abbreviations: TP, true positive

[a] Percent of total charts reviewed
[b] Percent of charts with true-positive disclosures, excluding undetermined cases

eTable 6. Timing of documented sexual trauma in relation to mental health diagnoses in the VUMC EHR

Timing of trauma	Schizophrenia			Bipolar disorder			MDD		
	N	% Total[a]	% TP-UD[b]	N	% Total[a]	% TP-UD[b]	N	% Total[a]	% TP-UD[b]
Trauma before diagnosis[c]	19	76.0%	90.5%	19	76.0%	82.6%	20	80.0%	95.2%
Diagnosis before trauma[d]	2	8.0%	9.5%	4	16.0%	17.4%	1	4.0%	4.8%
Undetermined	1	4.0%		2	8.0%		2	8.0%	
Total TP disclosures (not undetermined)	21	84.0%	100%	23	92.0%	100%	21	84.0%	100%
Total TP disclosures	22	88.0%		25	100%		23	92.0%	
Grand total	25	100%		25	100%		25	100%	

Abbreviations: TP, true positive; UD, undetermined; MDD, major depressive disorder

[a] Percent of total charts reviewed
[b] Percent of charts with true-positive disclosures, excluding undetermined cases
[c] The sexual trauma referred to in the notes likely occurred prior to the mental health diagnosis
[d] The mental health diagnosis likely occurred prior to the sexual trauma referred to in the notes

© 2024 Lake AM et al. *JAMA Psychiatry.*

eFigure 1. Data entry tool used for chart review determining the timing of the reported trauma in relation to the mental health diagnosis

GRID

* must provide value

Outcome diagnosis

* must provide value

Reviewer ID

* must provide value

Referring to the information documented in the chart, please identify to the extent possible the relative timing of the sexual assault disclosure and the mental health diagnosis (schizophrenia spectrum, bipolar disorder, or major depressive disorder). Sexual assault disclosures were defined using matches to keywords described in the attached PDF. Outcome diagnoses were determined using phecodes.

Keywords to search for to help with identifying mentions of the mental health diagnoses:

- **Schizophrenia:**
  - Schizophrenia
  - Schizophreniform
  - Schizoaffective
  - Psychosis
  - Psychotic
  - Delusions
  - Hallucinations
- **Bipolar disorder**
  - Mania
  - Manic
  - Pressured speech
- **Major depressive disorder**
  - MDD
  - Depressed / depression / depressive
  - Suicidal ideation

Lake SA Trauma Algorithm Definition v2 092723.pdf

* must provide value

- ○ The sexual trauma referred to in the note most likely preceded mental health diagnosis
- ○ Mental health diagnosis most likely preceded the sexual trauma referred to in the note
- ○ Not enough information to make a determination
- ○ "False positive" or indeterminate sexual trauma disclosure (examples: patient denies a history of sexual trauma, patient is reporting an assault that happened to someone else)

reset

Please provide a 1-3 sentence summary of your determination.

NOTE: No protected health information (PHI) should be recorded in this form.

* must provide value

© 2024 Lake AM et al. *JAMA Psychiatry.*

eTable 7. Genome-wide association studies used to generate polygenic scores

Phenotype	Reference	Accession	Ancestry group label	Sample size	Number of variants[a]	
					VUMC	*MGB*
Schizophrenia	Trubetskoy 2022[17]	Publicly available at pgc.unc.edu	European	53,386 cases<br>77,258 controls	786,374	1,106,302
			African[b]	6,152 cases<br>3,918 controls	774,688	N/A
Bipolar disorder	Mullins 2021[11]	Publicly available at pgc.unc.edu	European	41,917 cases<br>371,549 controls	785,475	1,105,253
	Bigdeli 2020[18]	Correspondence with author (TB Bigdeli)	African[b]	3,027 cases<br>7,988 controls	917,729	N/A
Depression	Als 2023[12]	Publicly available at ipsych.dk/en	European	294,322 cases<br>741,438 controls	785,280	1,102,343
	Levey 2021[19]	dbGaP accession: phs001672.v11.p1	African[b]	25,843 cases<br>33,757 controls	912,403	N/A

Abbreviations: VUMC, Vanderbilt University Medical Center; MGB, Mass General Brigham; CI, confidence interval.

[a] Number of variants included in polygenic scores after filtering summary statistics and intersecting with quality-controlled target sample variants at each site. At MGB, all variants in each discovery genome-wide association study were used. At VUMC, only variants with minor allele frequency >1% and imputation information score of >0.3 (when this field was available) in the discovery population were used in generating the polygenic scores.
[b] These polygenic scores were generated for target samples at VUMC only.

© 2024 Lake AM et al. *JAMA Psychiatry.*

eTable 8. Diagnosis codes relating to housing instability

Code	Description	Vocabulary
V60	Housing, household, and economic circumstances	ICD9CM
V60.0	Lack of housing	ICD9CM
V60.1	Inadequate housing	ICD9CM
V60.8	Other specified housing or economic circumstances	ICD9CM
V60.89	Other specified housing or economic circumstances	ICD9CM
V60.9	Unspecified housing or economic circumstance	ICD9CM
Z59.0	Homelessness	ICD10CM
Z59.00	Homelessness unspecified	ICD10CM
Z59.01	Sheltered homelessness	ICD10CM
Z59.02	Unsheltered homelessness	ICD10CM
Z59.1	Inadequate housing	ICD10CM
Z59.8	Other problems related to housing and economic circumstances	ICD10CM
Z59.819	Housing instability, housed unspecified	ICD10CM
Z59.89	Other problems related to housing and economic circumstances	ICD10CM
Z59.9	Problem related to housing and economic circumstances, unspecified	ICD10CM

© 2024 Lake AM et al. *JAMA Psychiatry.*

eFigure 2. Site-specific and meta-analysis odds ratios for association between each mental health condition and its respective dichotomized polygenic score, stratified into those with or without sexual trauma disclosures

Phenotype and cohort	OR (95% CI)	No. total	No. with diagnosis	Interaction P–value
**Schizophrenia**				
VUMC (1KG–EU–clustered)	2.67 (2.04–3.49)	57,293	217	0.01
	1.21 (0.68–2.15)	826	57	
MGB (1KG–EU–clustered)	2.78 (2.09–3.69)	25,842	196	0.12
	1.64 (0.95–2.85)	734	59	
Meta–analysis (1KG–EU–clustered)	2.72 (2.23–3.31)	83,135	413	0.005 (0.01)
	1.42 (0.96–2.11)	1,560	116	
VUMC (1KG–YRI–clustered)	1.43 (1.00–2.03)	10,770	159	0.99
	1.28 (0.50–3.27)	232	23	
**Bipolar disorder**				
VUMC (1KG–EU–clustered)	1.65 (1.48–1.83)	56,431	1,689	0.13
	1.18 (0.85–1.63)	777	277	
MGB (1KG–EU–clustered)	1.82 (1.61–2.06)	25,528	1,175	<0.001
	0.96 (0.69–1.35)	691	274	
Meta–analysis (1KG–EU–clustered)	1.72 (1.58–1.86)	81,959	2,864	<0.001 (0.002)
	1.07 (0.85–1.35)	1,468	551	
**Major depressive disorder**				
VUMC (1KG–EU–clustered)	1.34 (1.26–1.42)	54,719	5,534	0.93
	1.33 (0.98–1.80)	751	431	
MGB (1KG–EU–clustered)	1.34 (1.25–1.44)	24,546	5,300	0.84
	1.33 (0.89–1.98)	704	549	
Meta–analysis (1KG–EU–clustered)	1.34 (1.28–1.40)	79,265	10,834	0.85 (1.0)
	1.33 (1.04–1.69)	1,455	980	
VUMC (1KG–YRI–clustered)	1.21 (1.04–1.41)	10,317	912	0.89
	1.27 (0.68–2.38)	210	114	

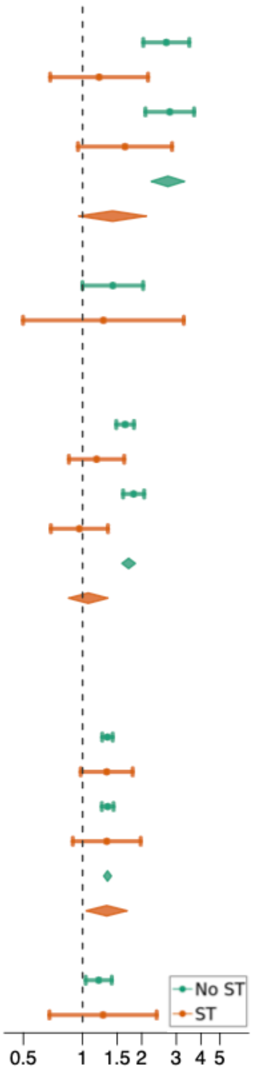

Each polygenic score is dichotomized at the 75th percentile. Multiplicative GxE interaction p-values for each condition are shown, with Bonferroni-adjusted (N=3) p-values included for each meta-analysis. All models are adjusted for EHR-median age, EHR-recorded sex, and the first three principal components estimated from genetic data. ST, sexual trauma; CI, confidence interval; VUMC, Vanderbilt University Medical Center; MGB, Mass General Brigham.

© 2024 Lake AM et al. *JAMA Psychiatry.*

eTable 11. GxE interaction results on the additive scale with polygenic score dichotomized at the 75th percentile within each study population

Phenotype and Cohort		Odds ratio (95% CI)[a]		RERI (95% CI)
		No ST	ST	
**Schizophrenia**				
VUMC (1KG-EU-clustered)	$PGS\text{-}SCZ_{75} = 0$	1.0	23.02 (15.44, 34.31)	
	$PGS\text{-}SCZ_{75} = 1$	2.67 (2.04, 3.49)	27.9 (16.77, 46.42)	3.22 (-11.76, 18.19)
MGB (1KG-EU-clustered)	$PGS\text{-}SCZ_{75} = 0$	1.0	13.66 (9.02, 20.68)	
	$PGS\text{-}SCZ_{75} = 1$	2.75 (2.07, 3.66)	23.2 (14.56, 36.97)	7.79 (-3.12, 18.70)
VUMC (1KG-YRI-clustered)	$PGS\text{-}SCZ_{75} = 0$	1.0	8.53 (4.79, 15.21)	
	$PGS\text{-}SCZ_{75} = 1$	1.38 (0.97, 1.96)	11.8 (5.42, 25.70)	2.90 (-7.06, 12.85)
**Bipolar disorder**				
VUMC (1KG-EU-clustered)	$PGS\text{-}BD_{75} = 0$	1.0	14.78 (12.16, 17.97)	
	$PGS\text{-}BD_{75} = 1$	1.63 (1.47, 1.81)	18.70 (14.31, 24.42)	3.28 (-2.24, 8.80)
MGB (1KG-EU-clustered)	$PGS\text{-}BD_{75} = 0$	1.0	14.85 (12.12, 18.21)	
	$PGS\text{-}BD_{75} = 1$	1.82 (1.60, 2.05)	14.54 (10.95, 19.32)	-1.13 (-5.99, 3.73)
**Major depressive disorder**				
VUMC (1KG-EU-clustered)	$PGS\text{-}MDD_{75} = 0$	1.0	10.44 (8.63, 12.62)	
	$PGS\text{-}MDD_{75} = 1$	1.34 (1.26, 1.42)	14.16 (11.16, 17.97)	3.39 (-0.46, 7.23)
MGB (1KG-EU-clustered)	$PGS\text{-}MDD_{75} = 0$	1.0	11.28 (9.08, 14.00)	
	$PGS\text{-}MDD_{75} = 1$	1.34 (1.25, 1.44)	15.77 (11.29, 22.03)	4.15 (-1.61, 9.91)
VUMC (1KG-YRI-clustered)	$PGS\text{-}MDD_{75} = 0$	1.0	11.44 (8.17, 16.03)	
	$PGS\text{-}MDD_{75} = 1$	1.21 (1.04, 1.41)	14.47 (8.59, 24.37)	2.82 (-5.5, 11.13)

Abbreviations: VUMC, Vanderbilt University Medical Center; MGB, Mass General Brigham; $PGS\text{-}SCZ_{75}$, dichotomized schizophrenia PGS; $PGS\text{-}BD_{75}$, dichotomized bipolar disorder PGS; $PGS\text{-}MDD_{75}$, dichotomized major depressive disorder PGS; RERI, Relative Excess Risk due to Interaction; CI, confidence interval.

[a] Odds ratios are reported for each of the exposure groups using a single reference category. All models are adjusted for EHR-median age, EHR-recorded sex, and the first three principal components estimated from genetic data.

© 2024 Lake AM et al. *JAMA Psychiatry.*

eFigure 3. Goodness of fit (pseudo-R^2) statistics for each regression model in the main GxE interaction analysis

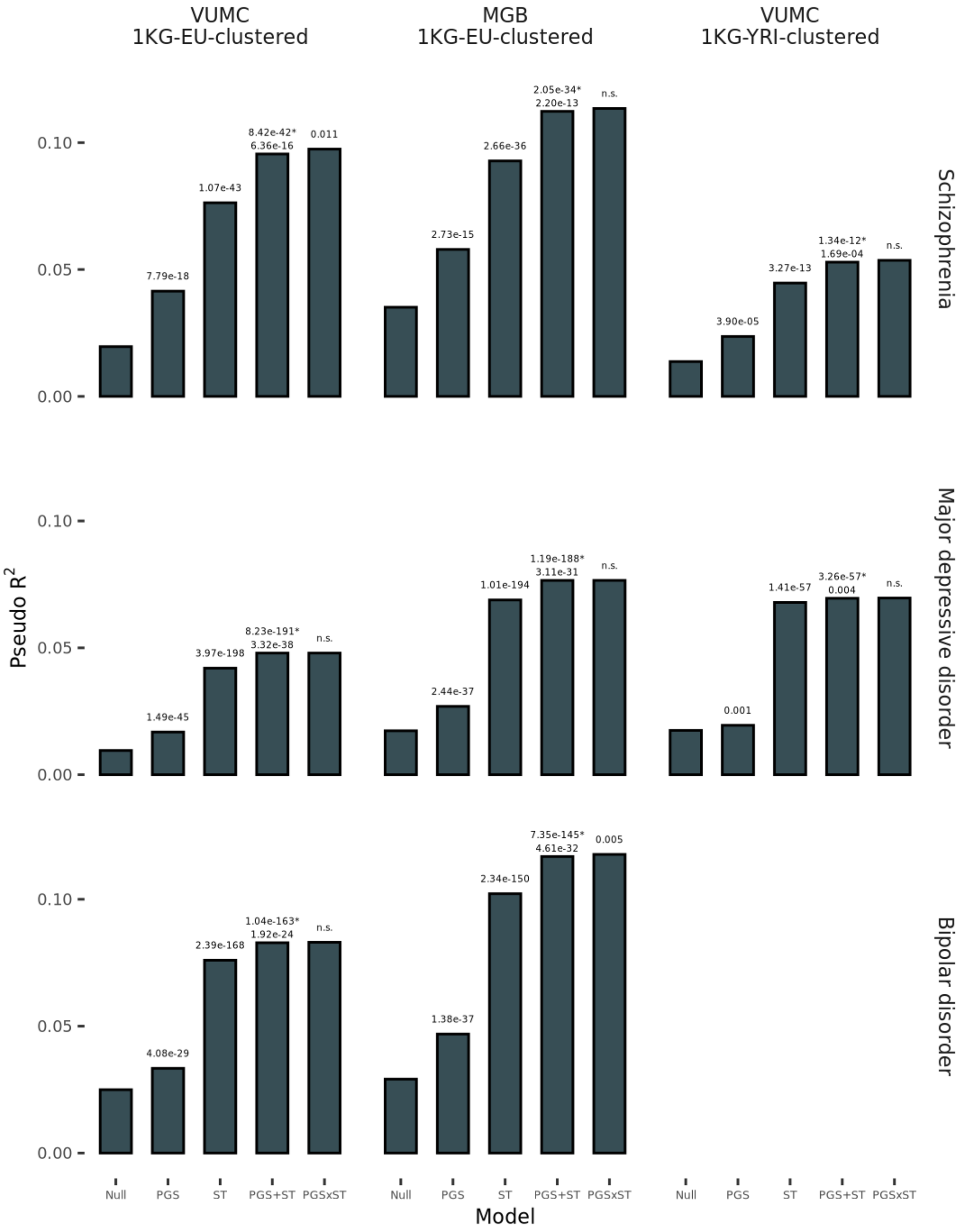

Likelihood ratio test p-values are shown above each bar. PGS and sexual trauma (ST) models are compared against the nested covariates-only (null) model. The PGS+ST model is compared against the PGS (*) and ST models. Finally, the PGSxST model is compared against the nested PGS+ST model. The "null" model includes EHR-median age, EHR-recorded sex, and the first three genotyping principal components. PGS, polygenic score; ST, sexual trauma; VUMC, Vanderbilt University Medical Center; MGB, Mass General Brigham.

© 2024 Lake AM et al. *JAMA Psychiatry.*

eTable 13. Main effect associations between sexual trauma history and mental health diagnoses after adjusting for comorbid mental health diagnoses

Phenotype and Cohort	No. total	No. with diagnosis	Odds Ratio (95% CI)[a]	P-value
**Schizophrenia**				
VUMC (1KG-EU-clustered)	58,119	274	3.19 (2.24-4.54)	<0.001
MGB (1KG-EU-clustered)	26,576	255	2.72 (1.93-3.85)	<0.001
VUMC (1KG-YRI-clustered)	11,002	182	2.36 (1.38-4.06)	0.002
**Bipolar disorder**				
VUMC (1KG-EU-clustered)	57,208	1,966	6.19 (5.16-7.43)	<0.001
MGB (1KG-EU-clustered)	26,219	1,449	4.92 (4.08-5.93)	<0.001
VUMC (1KG-YRI-clustered)	10,850	405	5.77 (4.07-8.19)	<0.001
**Major depressive disorder**				
VUMC (1KG-EU-clustered)	55,470	5,965	7.05 (5.99-8.30)	<0.001
MGB (1KG-EU-clustered)	25,250	5,849	7.72 (6.36-9.37)	<0.001
VUMC (1KG-YRI-clustered)	10,527	1,026	7.47 (5.49-10.17)	<0.001

Abbreviations: VUMC, Vanderbilt University Medical Center; MGB, Mass General Brigham; CI, confidence interval.

[a] All models are adjusted for EHR-median age, EHR-recorded sex, and the presence of the other two diagnoses (e.g., models for schizophrenia include covariates for both bipolar disorder and major depressive disorder diagnosis).

© 2024 Lake AM et al. *JAMA Psychiatry.*

eFigure 4. GxE interaction results from the comorbid mental health condition sensitivity analysis

Phenotype and cohort	OR (95% CI)	No. total	No. with diagnosis	Interaction P–value
**Schizophrenia**				
VUMC (1KG–EU–clustered)	1.71 (1.46–2.00)	57,293	217	0.01
	1.15 (0.86–1.53)	826	57	
MGB (1KG–EU–clustered)	1.56 (1.34–1.83)	25,842	196	0.51
	1.31 (0.98–1.75)	734	59	
Meta–analysis (1KG–EU–clustered)	1.63 (1.46–1.83)	83,135	413	0.03
	1.22 (1.00–1.50)	1,560	116	
**Bipolar disorder**				
VUMC (1KG–EU–clustered)	1.27 (1.21–1.34)	56,431	1,689	0.09
	1.03 (0.88–1.21)	777	277	
MGB (1KG–EU–clustered)	1.34 (1.26–1.44)	25,528	1,175	0.14
	1.14 (0.96–1.35)	691	274	
Meta–analysis (1KG–EU–clustered)	1.30 (1.25–1.35)	81,959	2,864	0.03
	1.08 (0.96–1.21)	1,468	551	

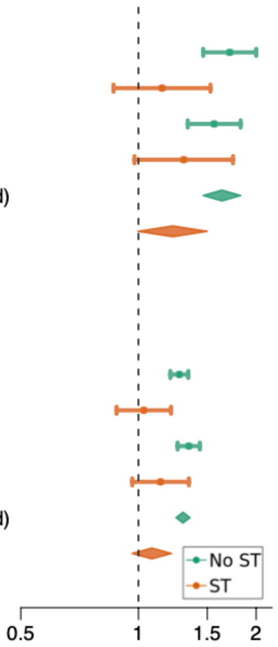

Only mental health conditions with a significant interaction effect in the main analysis are analyzed here. Mental health odds ratios per standard deviation unit increase in polygenic score, stratified into those with or without sexual trauma disclosures, are shown, alongside p-values from the multiplicative interaction test. All models are adjusted for EHR-median age, EHR-recorded sex, and the first three principal components estimated from genetic data. The model for each condition was additionally adjusted for the two other conditions (e.g., the schizophrenia model was adjusted for the presence of comorbid bipolar disorder and depression diagnoses). VUMC, Vanderbilt University Medical Center; MGB, Mass General Brigham; CI, confidence interval.

© 2024 Lake AM et al. *JAMA Psychiatry.*

eFigure 5. Goodness of fit (pseudo-R^2) statistics for each regression model after adjusting for comorbid mental health conditions

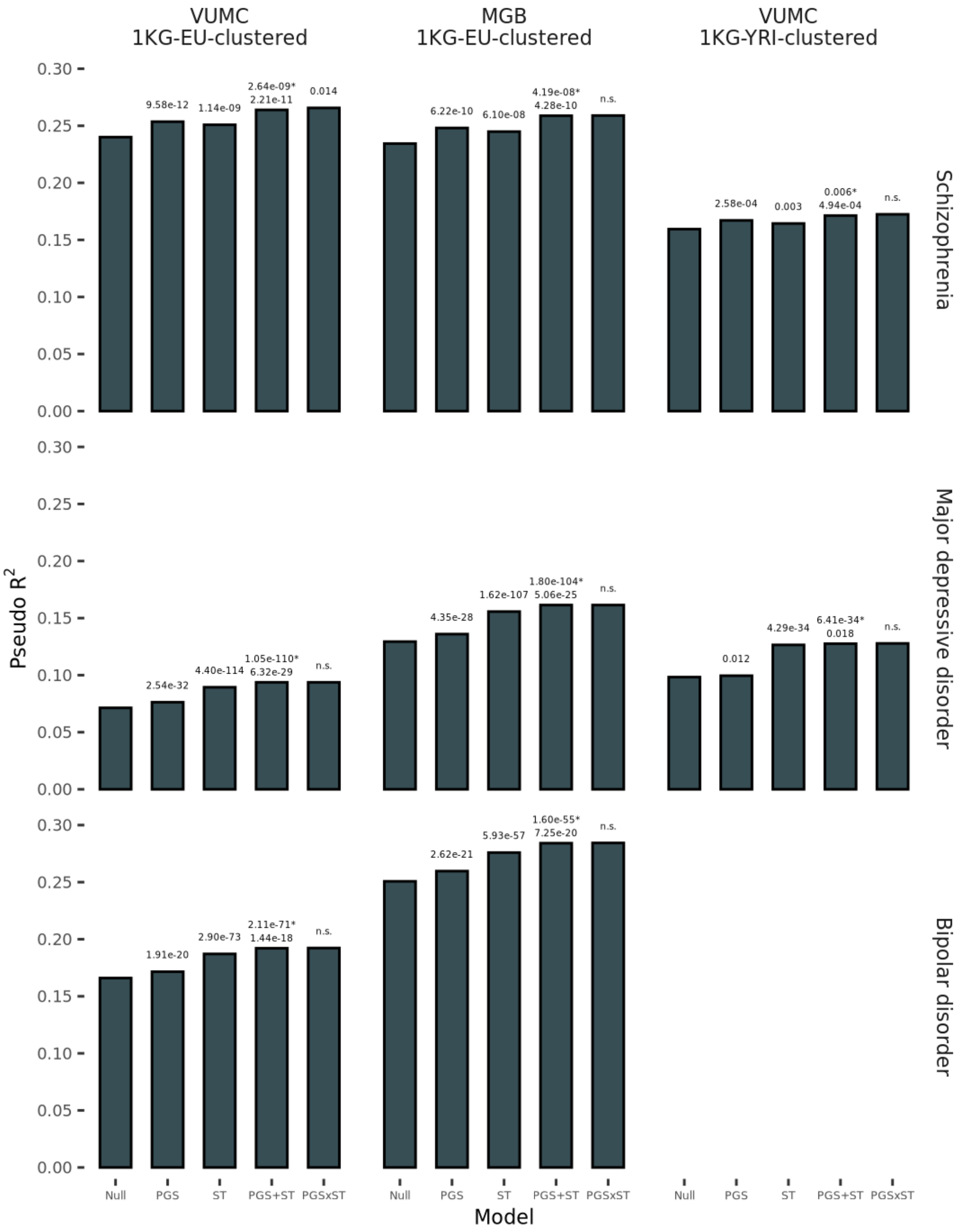

Likelihood ratio test p-values are shown above each bar. PGS and sexual trauma (ST) models are compared against the nested covariates-only (null) model. The PGS+ST model is compared against the PGS (*) and ST models. Finally, the PGSxST model is compared against the nested PGS+ST model. The "null" model includes EHR-median age, EHR-recorded sex, the first three genotyping principal components, and the presence of the other two diagnoses (e.g., models for schizophrenia include covariates for both bipolar disorder and major depressive disorder diagnosis). PGS, polygenic score; ST, sexual trauma; VUMC, Vanderbilt University Medical Center; MGB, Mass General Brigham.

© 2024 Lake AM et al. *JAMA Psychiatry.*

eTable 14. Associations between sexual trauma history and polygenic scores

Phenotype and Cohort	No. total	Odds ratio (95% CI)[a]	P-value
**Schizophrenia**			
VUMC (1KG-EU-clustered)	58,119	1.21 (1.13-1.31)	<0.001
MGB (1KG-EU-clustered)	26,576	1.23 (1.14-1.33)	<0.001
VUMC (1KG-YRI-clustered)	11,002	1.35 (1.16-1.56)	<0.001
**Bipolar disorder**			
VUMC (1KG-EU-clustered)	57,208	1.26 (1.17-1.36)	<0.001
MGB (1KG-EU-clustered)	26,219	1.27 (1.17-1.37)	<0.001
**Major depressive disorder**			
VUMC (1KG-EU-clustered)	55,470	1.38 (1.28-1.49)	<0.001
MGB (1KG-EU-clustered)	25,250	1.30 (1.20-1.40)	<0.001
VUMC (1KG-YRI-clustered)	10,527	1.13 (0.98-1.29)	0.09

Abbreviations: VUMC, Vanderbilt University Medical Center; MGB, Mass General Brigham; CI, confidence interval.

[a] All models are adjusted for EHR-median age, EHR-recorded sex, and the first three principal components estimated from genetic data.

© 2024 Lake AM et al. *JAMA Psychiatry.*

eFigure 6. Results of simulation analyses

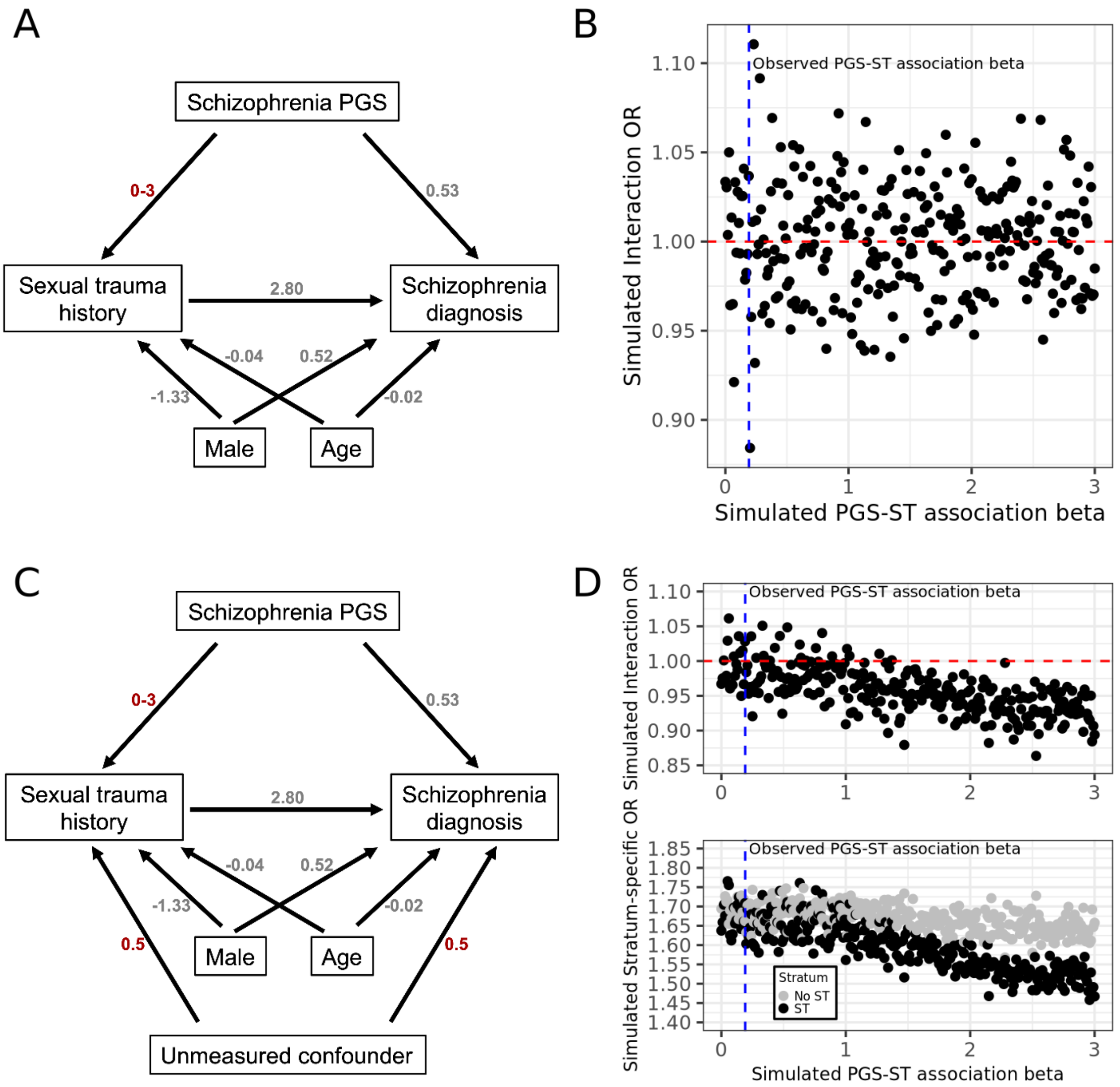

Simulated datasets of 1 million individuals each were generated to test the effects of polygenic score-trauma (PGS-ST) correlation and confounding bias on GxE interaction analysis findings. **A)** Directed acyclic graph (DAG) with prespecified causal relationships used to simulate (using simDAG[16]) the impact of increasing PGS-ST correlation on the GxE interaction effect. Each causal arrow is labeled with its respective effect size (log odds ratio) used to generate the simulated dataset. Effect sizes in grey were derived directly from schizophrenia GxE interaction regression analysis presented in **Figure 2** of the main text. **B)** Results of GxE interaction test run on each simulated dataset generated using the DAG in **panel A**. **C)** DAG used to simulate the impact of increasing PGS-ST correlation on the GxE interaction effect estimates, in the presence of an unmeasured confounder of trauma and schizophrenia. **D)** Results of GxE interaction test run on each simulated dataset generated by the DAG in **panel C**. Both interaction effect estimates (upper panel) and trauma-stratified PGS effect estimates (lower panel) are presented.

© 2024 Lake AM et al. *JAMA Psychiatry.*

eTable 15. Procedure codes used for identifying psychiatry visits

Code	Description	Vocabulary
90785	Interactive complexity (List separately in addition to the code for primary procedure)	CPT4
90791	Psychiatric diagnostic evaluation	CPT4
90792	Psychiatric diagnostic evaluation with medical services	CPT4
90801	Psychiatric diagnostic interview examination	CPT4
90804	Individual psychotherapy, insight oriented, behavior modifying and/or supportive, in an office or outpatient facility, approximately 20 to 30 minutes face-to-face with the patient	CPT4
90805	Individual psychotherapy, insight oriented, behavior modifying and/or supportive, in an office or outpatient facility, approximately 20 to 30 minutes face-to-face with the patient; with medical evaluation and management services	CPT4
90806	Individual psychotherapy, insight oriented, behavior modifying and/or supportive, in an office or outpatient facility, approximately 45 to 50 minutes face-to-face with the patient	CPT4
90807	Individual psychotherapy, insight oriented, behavior modifying and/or supportive, in an office or outpatient facility, approximately 45 to 50 minutes face-to-face with the patient; with medical evaluation and management services	CPT4
90832	Psychotherapy, 30 minutes with patient	CPT4
90833	Psychotherapy, 30 minutes with patient when performed with an evaluation and management service (List separately in addition to the code for primary procedure)	CPT4
90834	Psychotherapy, 45 minutes with patient	CPT4
90836	Psychotherapy, 45 minutes with patient when performed with an evaluation and management service (List separately in addition to the code for primary procedure)	CPT4
90837	Psychotherapy, 60 minutes with patient	CPT4
90838	Psychotherapy, 60 minutes with patient when performed with an evaluation and management service (List separately in addition to the code for primary procedure)	CPT4
90839	Psychotherapy for crisis; first 60 minutes	CPT4
90840	Psychotherapy for crisis; each additional 30 minutes (List separately in addition to code for primary service)	CPT4
90845	Psychoanalysis	CPT4
90846	Family psychotherapy (without the patient present), 50 minutes	CPT4
90847	Family psychotherapy (conjoint psychotherapy) (with patient present), 50 minutes	CPT4
90849	Multiple-family group psychotherapy	CPT4
90853	Group psychotherapy (other than of a multiple-family group)	CPT4
90857	Interactive group psychotherapy	CPT4
90862	Pharmacologic management, including prescription, use, and review of medication with no more than minimal medical psychotherapy	CPT4
90863	Pharmacologic management, including prescription and review of medication, when performed with psychotherapy services (List separately in addition to the code for primary procedure)	CPT4

© 2024 Lake AM et al. *JAMA Psychiatry.*

90865	Narcosynthesis for psychiatric diagnostic and therapeutic purposes (eg, sodium amobarbital (Amytal) interview)	CPT4
90867	Therapeutic repetitive transcranial magnetic stimulation (TMS) treatment; initial, including cortical mapping, motor threshold determination, delivery and management	CPT4
90868	Therapeutic repetitive transcranial magnetic stimulation (TMS) treatment; subsequent delivery and management, per session	CPT4
90869	Therapeutic repetitive transcranial magnetic stimulation (TMS) treatment; subsequent motor threshold re-determination with delivery and management	CPT4
90870	Electroconvulsive therapy (includes necessary monitoring)	CPT4
90875	Individual psychophysiological therapy incorporating biofeedback training by any modality (face-to-face with the patient), with psychotherapy (eg, insight oriented, behavior modifying or supportive psychotherapy); 30 minutes	CPT4
90876	Individual psychophysiological therapy incorporating biofeedback training by any modality (face-to-face with the patient), with psychotherapy (eg, insight oriented, behavior modifying or supportive psychotherapy); approximately 45-50 minutes	CPT4
90880	Hypnotherapy	CPT4
90885	Psychiatric evaluation of hospital records, other psychiatric reports, psychometric and/or projective tests, and other accumulated data for medical diagnostic purposes	CPT4
90887	Interpretation or explanation of results of psychiatric, other medical examinations and procedures, or other accumulated data to family or other responsible persons, or advising them how to assist patient	CPT4
90889	Preparation of report of patient's psychiatric status, history, treatment, or progress (other than for legal or consultative purposes) for other individuals, agencies, or insurance carriers	CPT4
90899	Unlisted psychiatric service or procedure	CPT4
95970<br>95974<br>95975	Electronic analysis of implanted neurostimulator pulse generator/transmitter	CPT4
M0064	Brief office visit for the sole purpose of monitoring or changing drug prescriptions used in the treatment of mental psychoneurotic and personality disorders	CPT4
G0177	Training and educational services related to the care and treatment of patient's disabling mental health problems per session (45 minutes or more)	HCPCS
G0410	Group psychotherapy other than of a multiple-family group, in a partial hospitalization setting, approximately 45 to 50 minutes	HCPCS
H0002	Behavioral health screening to determine eligibility for admission to treatment program	HCPCS
H0031	Mental health assessment, by non-physician	HCPCS
H0035	Mental health partial hospitalization, treatment, less than 24 hours	HCPCS
H0036	Community psychiatric supportive treatment, face-to-face, per 15 minutes	HCPCS
S9480	Intensive outpatient psychiatric services, per diem	HCPCS
GZ3ZZZZ	Medication Management	ICD10PCS
GZB0ZZZ	Electroconvulsive Therapy, Unilateral-Single Seizure	ICD10PCS
GZB2ZZZ	Electroconvulsive Therapy, Bilateral-Single Seizure	ICD10PCS
94.27	Other electroshock therapy	ICD9Proc

© 2024 Lake AM et al. *JAMA Psychiatry.*

eTable 16. Results of mapping sexual trauma disclosure notes to clinical settings

Clinical setting	VUMC<br>N=1,082<br>N (% of cases)	MGB<br>N=752<br>N (% of cases)
Psychiatry[a]	343 (32%)	235 (31%)
Primary care	114 (11%)	
Emergency medicine	71 (7%)	
Obstetrics and gynecology	67 (6%)	

Abbreviations: VUMC, Vanderbilt University Medical Center; MGB, Mass General Brigham.

[a] Psychiatry visits were identified by linking each patient's earliest sexual trauma disclosure note to psychiatry procedure codes (both sites) or to specialty-labeled clinical encounters (VUMC only). Visits corresponding to other specialties were identified using specialty-labeled clinical encounters, available at VUMC only (see **eMethods**).

eTable 17. Main effect associations between sexual trauma history and mental health diagnoses after removing sexual trauma cases whose initial disclosures were reported in a psychiatry setting (clinical setting sensitivity analysis)

Phenotype and Cohort	No. total	No. with diagnosis	Odds Ratio (95% CI)[a]	P-value
**Schizophrenia**				
VUMC (1KG-EU-clustered)	57,854	238	8.70 (5.41-13.99)	<0.001
MGB (1KG-EU-clustered)	26,347	246	14.96 (10.65-21.01)	<0.001
VUMC (1KG-YRI-clustered)	10,933	174	7.93 (4.49-14.00)	<0.001
**Bipolar disorder**				
VUMC (1KG-EU-clustered)	56,967	1,842	9.77 (8.01-11.93)	<0.001
MGB (1KG-EU-clustered)	26,007	1,356	11.36 (9.31-13.86)	<0.001
VUMC (1KG-YRI-clustered)	10,784	379	11.08 (7.64-16.07)	<0.001
**Major depressive disorder**				
VUMC (1KG-EU-clustered)	55,226	5,789	8.04 (6.73-9.61)	<0.001
MGB (1KG-EU-clustered)	25,029	5,657	9.19 (7.47-11.30)	<0.001
VUMC (1KG-YRI-clustered)	10,468	981	8.25 (5.93-11.49)	<0.001

Abbreviations: VUMC, Vanderbilt University Medical Center; MGB, Mass General Brigham; CI, confidence interval.

[a] All models are adjusted for EHR-median age and EHR-recorded sex.

eFigure 7. GxE interaction results from the clinical setting sensitivity analysis

Phenotype and cohort	OR (95% CI)	No. total	No. with diagnosis	Interaction P–value
**Schizophrenia**				
VUMC (1KG–EU–clustered)	1.87 (1.60–2.17)	57,293	217	0.06
	1.23 (0.77–1.96)	561	21	
MGB (1KG–EU–clustered)	1.76 (1.51–2.04)	25,842	196	0.02
	1.14 (0.84–1.54)	505	50	
Meta–analysis (1KG–EU–clustered)	1.81 (1.63–2.02)	83,135	413	0.002
	1.16 (0.90–1.50)	1,066	71	
**Bipolar disorder**				
VUMC (1KG–EU–clustered)	1.30 (1.24–1.37)	56,431	1,689	0.16
	1.09 (0.89–1.33)	536	153	
MGB (1KG–EU–clustered)	1.45 (1.36–1.54)	25,528	1,175	0.002
	1.05 (0.87–1.28)	479	181	
Meta–analysis (1KG–EU–clustered)	1.36 (1.31–1.42)	81,959	2,864	0.001
	1.07 (0.93–1.23)	1,015	334	

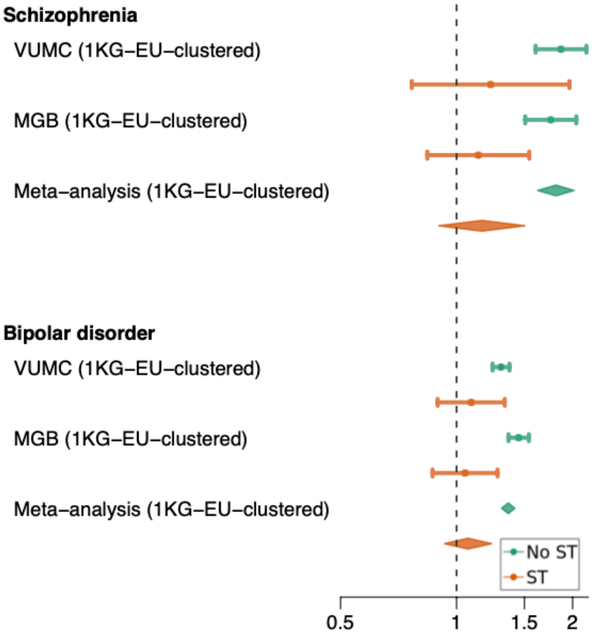

Mental health conditions with a significant interaction effect in the main analysis are analyzed here. Mental health odds ratios per standard deviation unit increase in polygenic score stratified into those with (ST) or without (no ST) sexual trauma disclosures, after removing sexual trauma cases from the analysis whose earliest disclosures were reported in a psychiatry setting, are shown, alongside p-values from the multiplicative interaction test. VUMC, Vanderbilt University Medical Center; MGB, Mass General Brigham; CI, confidence interval.

© 2024 Lake AM et al. *JAMA Psychiatry.*

eFigure 8. Goodness of fit (pseudo-R^2) statistics for each regression model in the clinical setting sensitivity analysis

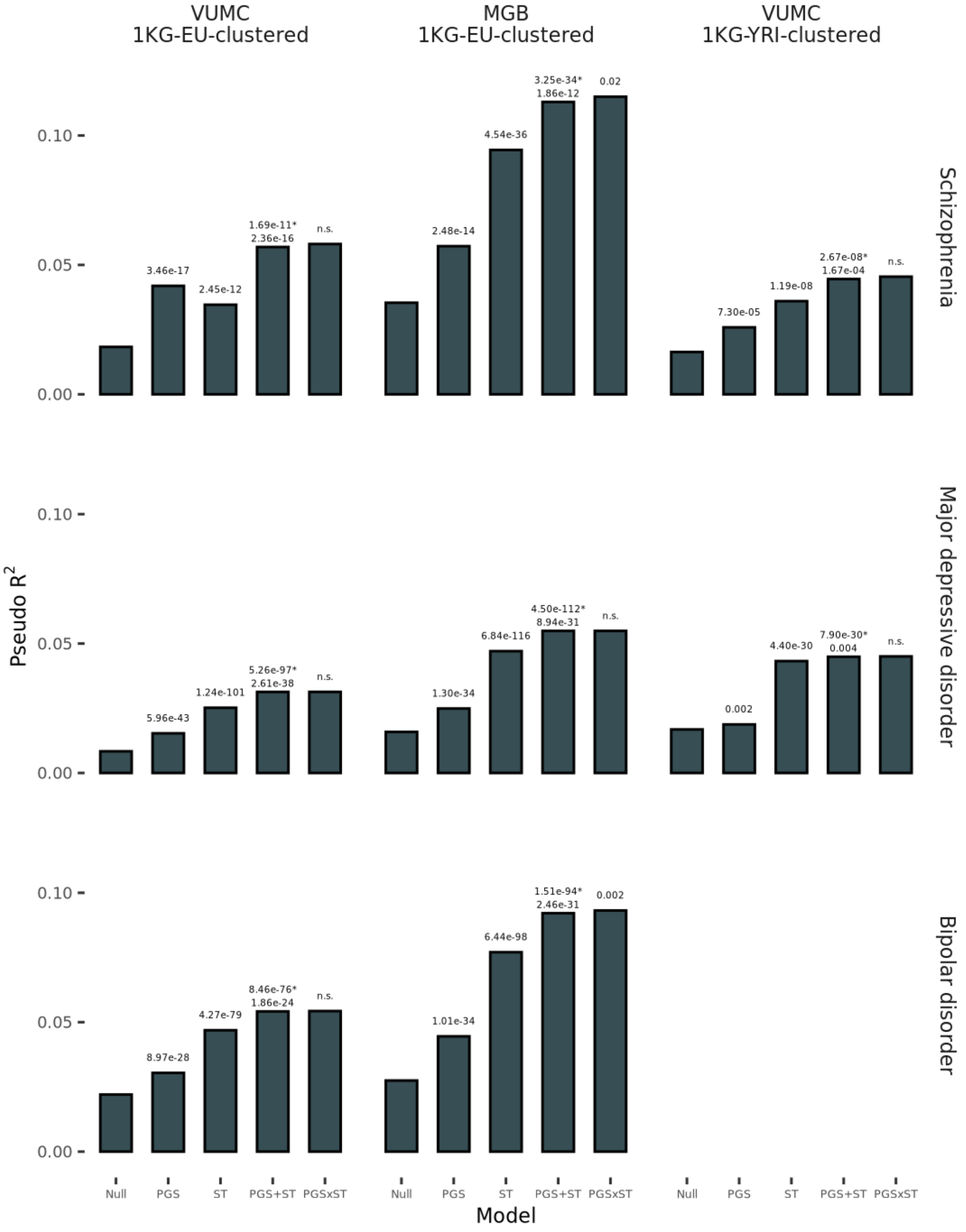

Sexual trauma cases whose initial disclosures were reported in a psychiatry setting were removed from this analysis. Likelihood ratio test p-values are shown above each bar. PGS and sexual trauma (ST) models are compared against the nested covariates-only (null) model. The PGS+ST model is compared against the PGS (*) and ST models. Finally, the PGSxST model is compared against the nested PGS+ST model. The “null” model includes EHR-median age, EHR-recorded sex, and the first three genotyping principal components. PGS, polygenic score; ST, sexual trauma; VUMC, Vanderbilt University Medical Center; MGB, Mass General Brigham.

© 2024 Lake AM et al. *JAMA Psychiatry.*

eTable 18. Main effect associations between sexual trauma history and mental health diagnoses after adjusting for substance use disorder diagnoses

Phenotype and Cohort	No. total	No. with diagnosis	Odds Ratio (95% CI)[a]	P-value
**Schizophrenia**				
VUMC (1KG-EU-clustered)	58,119	274	9.59 (6.86-13.40)	<0.001
MGB (1KG-EU-clustered)	26,576	255	6.16 (4.43-8.57)	<0.001
VUMC (1KG-YRI-clustered)	11,002	182	4.63 (2.83-7.58)	<0.001
**Bipolar disorder**				
VUMC (1KG-EU-clustered)	57,208	1,966	9.03 (7.59-10.75)	<0.001
MGB (1KG-EU-clustered)	26,219	1,449	7.73 (6.47-9.25)	<0.001
VUMC (1KG-YRI-clustered)	10,850	405	8.17 (5.83-11.45)	<0.001
**Major depressive disorder**				
VUMC (1KG-EU-clustered)	55,470	5,965	8.14 (6.94-9.55)	<0.001
MGB (1KG-EU-clustered)	25,250	5,849	8.11 (6.70-9.81)	<0.001
VUMC (1KG-YRI-clustered)	10,527	1,026	8.31 (6.13-11.25)	<0.001

Abbreviations: VUMC, Vanderbilt University Medical Center; MGB, Mass General Brigham; CI, confidence interval.

[a] All models are adjusted for EHR-median age, EHR-recorded sex, and the presence of a substance use disorder diagnosis.

© 2024 Lake AM et al. *JAMA Psychiatry.*

eTable 19. Main effect associations between sexual trauma history and mental health diagnoses after adjusting for billing codes relating to housing instability

Phenotype and Cohort	No. total	No. with diagnosis	Odds Ratio (95% CI)[a]	P-value
**Schizophrenia**				
VUMC (1KG-EU-clustered)	58,119	274	14.09 (10.11-19.63)	<0.001
MGB (1KG-EU-clustered)	26,576	255	6.28 (4.42-8.94)	<0.001
VUMC (1KG-YRI-clustered)	11,002	182	6.27 (3.79-10.39)	<0.001
**Bipolar disorder**				
VUMC (1KG-EU-clustered)	57,208	1,966	13.13 (11.16-15.44)	<0.001
MGB (1KG-EU-clustered)	26,219	1,449	9.8 (8.20-11.71)	<0.001
VUMC (1KG-YRI-clustered)	10,850	405	11.75 (8.54-16.17)	<0.001
**Major depressive disorder**				
VUMC (1KG-EU-clustered)	55,470	5,965	10.35 (8.91-12.03)	<0.001
MGB (1KG-EU-clustered)	25,250	5,849	10.10 (8.39-12.15)	<0.001
VUMC (1KG-YRI-clustered)	10,527	1,026	10.68 (8.01-14.22)	<0.001

Abbreviations: VUMC, Vanderbilt University Medical Center; MGB, Mass General Brigham; CI, confidence interval.

[a] All models are adjusted for EHR-median age, EHR-recorded sex, and the presence of a diagnosis billing code relating housing instability.

© 2024 Lake AM et al. *JAMA Psychiatry.*

eFigure 9. GxE interaction results from the substance use disorder sensitivity analysis

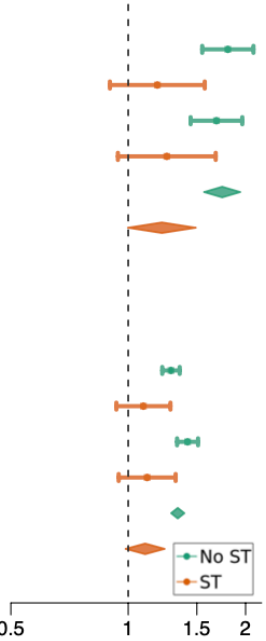

Phenotype and cohort	OR (95% CI)	No. total	No. with diagnosis	Interaction P–value
**Schizophrenia**				
VUMC (1KG–EU–clustered)	1.80 (1.55–2.09)	57,293	217	0.006
	1.19 (0.90–1.57)	826	57	
MGB (1KG–EU–clustered)	1.68 (1.45–1.96)	25,842	196	0.10
	1.26 (0.94–1.68)	734	59	
Meta–analysis (1KG–EU–clustered)	1.74 (1.56–1.94)	83,135	413	0.002
	1.22 (1.00–1.49)	1,560	116	
**Bipolar disorder**				
VUMC (1KG–EU–clustered)	1.29 (1.22–1.36)	56,431	1,689	0.16
	1.09 (0.93–1.28)	777	277	
MGB (1KG–EU–clustered)	1.42 (1.33–1.51)	25,528	1,175	0.01
	1.12 (0.95–1.32)	691	274	
Meta–analysis (1KG–EU–clustered)	1.34 (1.29–1.39)	81,959	2,864	0.006
	1.11 (0.98–1.24)	1,468	551	

Mental health conditions with a significant interaction effect in the main analysis are analyzed here. Mental health odds ratios per one standard deviation unit increase in polygenic score, stratified into those with (ST) or without (no ST) sexual trauma disclosures, after adjusting for the presence of a substance use disorder diagnosis, are shown, alongside p-values from the multiplicative interaction test. VUMC, Vanderbilt University Medical Center; MGB, Mass General Brigham; CI, confidence interval.

© 2024 Lake AM et al. *JAMA Psychiatry.*

eFigure 10. Goodness of fit (pseudo-R^2) statistics for each regression model in the substance use disorder sensitivity analysis

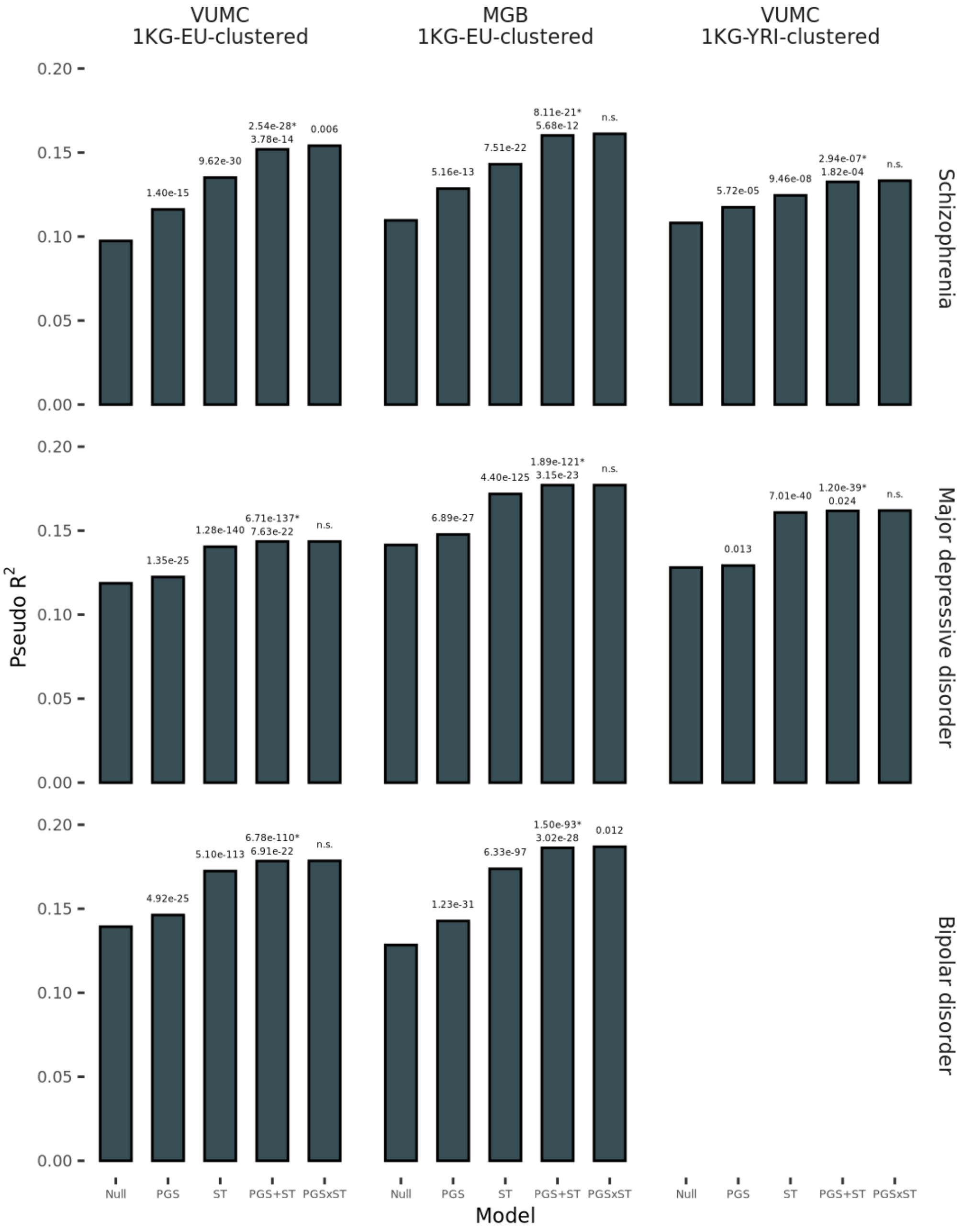

Likelihood ratio test p-values are shown above each bar. PGS and sexual trauma (ST) models are compared against the nested covariates-only (null) model. The PGS+ST model is compared against the PGS (*) and ST models. Finally, the PGSxST model is compared against the nested PGS+ST model. The "null" model includes the presence of a substance use disorder diagnosis (binary), EHR-median age, EHR-recorded sex, and the first three genotyping principal components. PGS, polygenic score; ST, sexual trauma; VUMC, Vanderbilt University Medical Center; MGB, Mass General Brigham.

© 2024 Lake AM et al. *JAMA Psychiatry.*

eFigure 11. GxE interaction results from the housing instability sensitivity analysis

Phenotype and cohort	OR (95% CI)	No. total	No. with diagnosis	Interaction P–value
**Schizophrenia**				
VUMC (1KG–EU–clustered)	1.88 (1.61–2.18)	57,293	217	0.003
	1.16 (0.87–1.55)	826	57	
MGB (1KG–EU–clustered)	1.69 (1.45–1.97)	25,842	196	0.15
	1.28 (0.95–1.71)	734	59	
Meta–analysis (1KG–EU–clustered)	1.78 (1.60–1.99)	83,135	413	0.002
	1.22 (0.99–1.49)	1,560	116	
**Bipolar disorder**				
VUMC (1KG–EU–clustered)	1.31 (1.24–1.37)	56,431	1,689	0.05
	1.07 (0.91–1.24)	777	277	
MGB (1KG–EU–clustered)	1.44 (1.35–1.53)	25,528	1,175	0.03
	1.15 (0.97–1.36)	691	274	
Meta–analysis (1KG–EU–clustered)	1.36 (1.31–1.41)	81,959	2,864	0.004
	1.10 (0.99–1.24)	1,468	551	

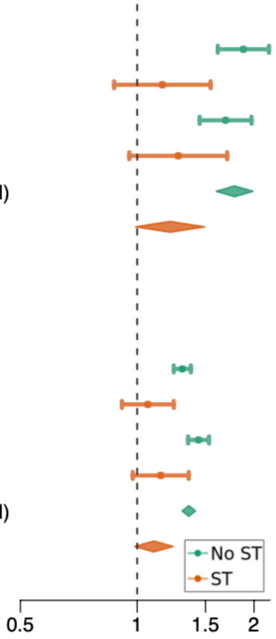

Mental health conditions with a significant interaction effect in the main analysis are analyzed here. Mental health odds ratios per one standard deviation unit increase in polygenic score, stratified into those with (ST) or without (no ST) sexual trauma disclosures, after adjusting for the presence of at least one housing instability billing code, are shown, alongside p-values from the multiplicative interaction test. VUMC, Vanderbilt University Medical Center; MGB, Mass General Brigham; CI, confidence interval.

© 2024 Lake AM et al. *JAMA Psychiatry.*

eFigure 12. Goodness of fit (pseudo-R^2) statistics for each regression model in the housing instability sensitivity analysis

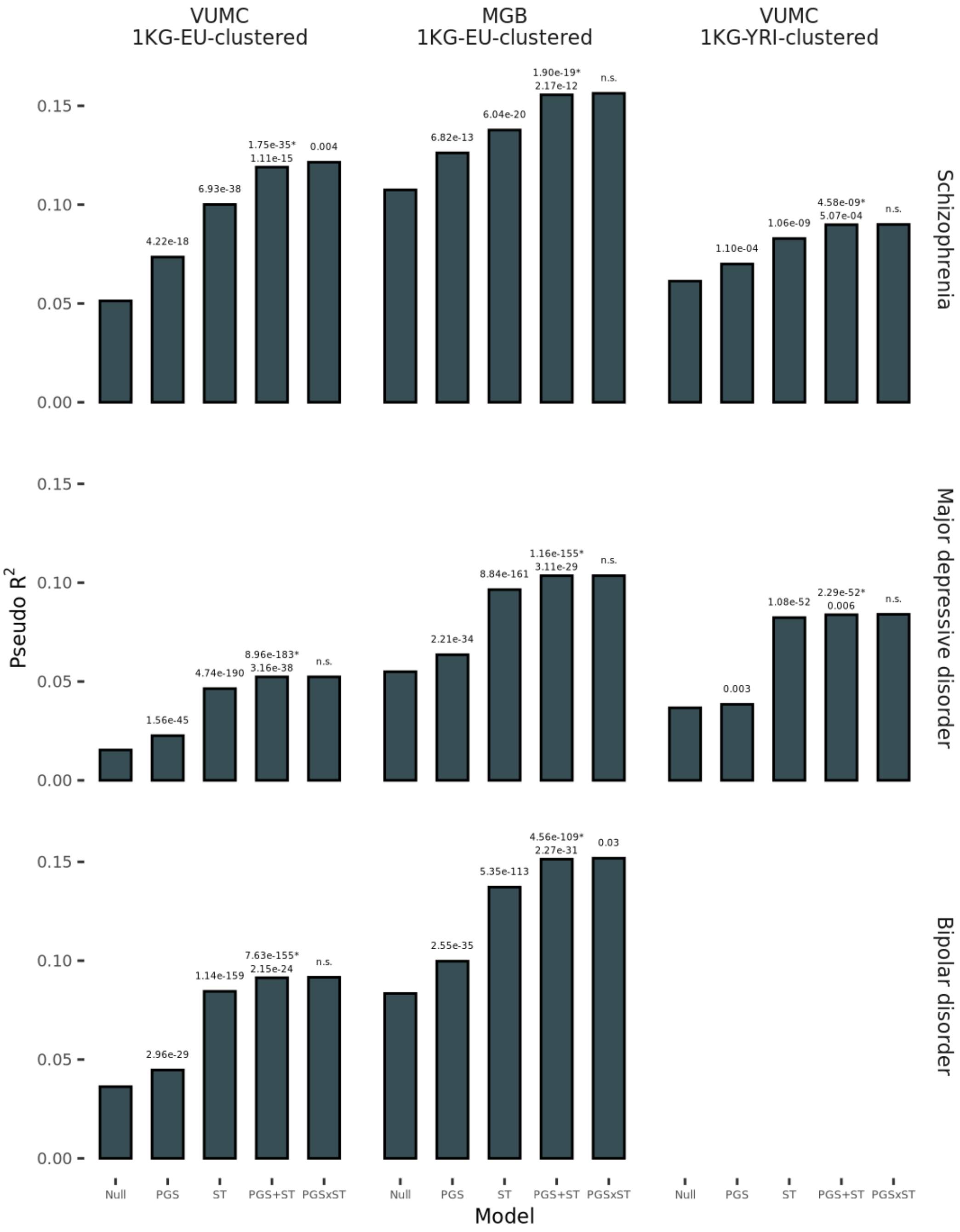

Likelihood ratio test p-values are shown above each bar. PGS and sexual trauma (ST) models are compared against the nested covariates-only (null) model. The PGS+ST model is compared against the PGS (*) and ST models. Finally, the PGSxST model is compared against the nested PGS+ST model. The “null” model includes the presence of a diagnosis code relating to housing instability (binary), EHR-median age, EHR-recorded sex, and the first three genotyping principal components as independent variables. PGS, polygenic score; ST, sexual trauma; VUMC, Vanderbilt University Medical Center; MGB, Mass General Brigham.

© 2024 Lake AM et al. *JAMA Psychiatry.*

eReferences

1. Roden D, Pulley J, Basford M, et al. Development of a large-scale de-identified DNA biobank to enable personalized medicine. *Clin Pharmacol Ther*. 2008;84(3):362-369. doi:10.1038/clpt.2008.89
2. Dennis JK, Sealock JM, Straub P, et al. Clinical laboratory test-wide association scan of polygenic scores identifies biomarkers of complex disease. *Genome Med*. 2021;13:6. doi:10.1186/s13073-020-00820-8
3. Abraham G, Qiu Y, Inouye M. FlashPCA2: principal component analysis of biobank-scale genotype datasets. *Bioinformatics*. 2017;33(17):2776-2778. doi:10.1093/bioinformatics/btx299
4. Auton A, Abecasis GR, Altshuler DM, et al. A global reference for human genetic variation. *Nature*. 2015;526(7571):68-74. doi:10.1038/nature15393
5. Das S, Forer L, Schönherr S, et al. Next-generation genotype imputation service and methods. Nat Genet. 2016;48(10):1284-1287. doi:10.1038/ng.3656
6. R Core Team. *R: A Language and Environment for Statistical Computing*. R Foundation for Statistical Computing; 2020. Accessed September 28, 2024. https://www.R-project.org/
7. Karlson EW, Boutin NT, Hoffnagle AG, Allen NL. Building the Partners HealthCare Biobank at Partners Personalized Medicine: informed consent, return of research results, recruitment lessons and operational considerations. J Pers Med. 2016;6(1):2. doi:10.3390/jpm6010002
8. Ge T, Chen CY, Ni Y, Feng YCA, Smoller JW. Polygenic prediction via Bayesian regression and continuous shrinkage priors. *Nat Commun*. 2019;10(1):1776. doi:10.1038/s41467-019-09718-5
9. Ruan Y, Lin YF, Feng YCA, et al. Improving polygenic prediction in ancestrally diverse populations. *Nat Genet*. 2022;54(5):573-580. doi:10.1038/s41588-022-01054-7
10. National Academies of Sciences, Engineering, and Medicine. *Using Population Descriptors in Genetics and Genomics Research: A New Framework for an Evolving Field*. The National Academies Press; 2023. doi:10.17226/26902
11. Mullins N, Forstner AJ, O'Connell KS, et al. Genome-wide association study of more than 40,000 bipolar disorder cases provides new insights into the underlying biology. *Nat Genet*. 2021;53(6):817-829. doi:10.1038/s41588-021-00857-4
12. Als TD, Kurki MI, Grove J, et al. Depression pathophysiology, risk prediction of recurrence and comorbid psychiatric disorders using genome-wide analyses. *Nat Med*. 2023;29(7):1832-1844. doi:10.1038/s41591-023-02352-1
13. Denny JC, Ritchie MD, Basford MA, et al. PheWAS: demonstrating the feasibility of a phenome-wide scan to discover gene–disease associations. *Bioinformatics*. 2010;26(9):1205-1210. doi:10.1093/bioinformatics/btq126
14. Carroll RJ, Bastarache L, Denny JC. R PheWAS: data analysis and plotting tools for phenome-wide association studies in the R environment. *Bioinformatics*. 2014;30(16):2375-2376. doi:10.1093/bioinformatics/btu197
15. Wu P, Gifford A, Meng X, et al. Mapping ICD-10 and ICD-10-CM codes to phecodes: workflow development and initial evaluation. *JMIR Med Inform*. 2019;7(4):e14325. doi:10.2196/14325
16. Denz R, Meiszl K. simDAG: Simulate data from a DAG and associated node information. Accessed March 29, 2024. https://cran.r-project.org/web/packages/simDAG/simDAG.pdf
17. Trubetskoy V, Pardiñas AF, Qi T, et al. Mapping genomic loci implicates genes and synaptic biology in schizophrenia. *Nature*. Published online April 8, 2022:1-13. doi:10.1038/s41586-022-04434-5
18. Bigdeli TB, Fanous AH, Li Y, et al. Genome-wide association studies of schizophrenia and bipolar disorder in a diverse cohort of US veterans. *Schizophr Bull*. 2020;47(2):517-529. doi:10.1093/schbul/sbaa133
19. Levey DF, Stein MB, Wendt FR, et al. Bi-ancestral depression GWAS in the Million Veteran Program and meta-analysis in >1.2 million subjects highlights new therapeutic directions. *Nat Neurosci*. 2021;24(7):954-963. doi:10.1038/s41593-021-00860-2
