# Supplementary material for: Sexual Trauma, Polygenic Scores, and Mental Health Diagnoses and Outcomes
Source: JAMA Psychiatry. 2024 Oct 30;82(1):75–84. doi: 10.1001/jamapsychiatry.2024.3426 (PMC11581726; doi:10.1001/jamapsychiatry.2024.3426)
Supplement: Supplement 3. — Data Sharing Statement [file jamapsychiatry-e243426-s003.pdf]

Data Sharing Statement

Lake. Sexual Trauma, Polygenic Scores, and Mental Health Diagnoses and Outcomes. *JAMA Psychiatry*. Published October 30, 2024. doi:10.1001/jamapsychiatry.2024.3426

Data
Data available: No

Additional Information
Explanation for why data not available: Data are available from Vanderbilt University Medical Center with institutional restrictions that apply to the acquisition, use, and dissemination of data. To request reasonable access to data for work conducted in a non-profit academic setting, please contact the Vanderbilt Institute for Clinical and Translational Research (research.support.services@vumc.org) and request an application to the Integrated Data Access and Services Core. Data from Mass General Brigham cannot be made publicly available due to IRB restrictions.
